# The nucleoid occlusion factor Noc controls DNA replication initiation in *Staphylococcus aureus*

**DOI:** 10.1371/journal.pgen.1006908

**Published:** 2017-07-19

**Authors:** Ting Pang, Xindan Wang, Hoong Chuin Lim, Thomas G. Bernhardt, David Z. Rudner

**Affiliations:** Department of Microbiology and Immunobiology, Harvard Medical School, Boston, MA, United States of America; University of Geneva Medical School, SWITZERLAND

## Abstract

Successive division events in the spherically shaped bacterium *Staphylococcus aureus* are oriented in three alternating perpendicular planes. The mechanisms that underlie this relatively unique pattern of division and coordinate it with chromosome segregation remain largely unknown. Thus far, the only known spatial regulator of division in this organism is the nucleoid occlusion protein Noc that inhibits assembly of the cytokinetic ring over the chromosome. However, Noc is not essential in *S*. *aureus*, indicating that additional regulators are likely to exist. To search for these factors, we screened for mutants that are synthetic lethal with Noc inactivation. Our characterization of these mutants led to the discovery that *S*. *aureus* Noc also controls the initiation of DNA replication. We show that cells lacking Noc over-initiate and mutations in the initiator gene *dnaA* suppress this defect. Importantly, these *dnaA* mutations also partially suppress the division problems associated with Δ*noc*. Reciprocally, we show that over-expression of DnaA enhances the over-initiation and cell division phenotypes of the Δ*noc* mutant. Thus, a single factor both blocks cell division over chromosomes and helps to ensure that new rounds of DNA replication are not initiated prematurely. This degree of economy in coordinating key cell biological processes has not been observed in rod-shaped bacteria and may reflect the challenges posed by the reduced cell volume and complicated division pattern of this spherical pathogen.

## Author summary

The mechanisms by which bacteria coordinate cell division with chromosome replication and segregation remain poorly understood. This coordination is particularly challenging in the spherical bacterium *Staphylococcus aureus*, which unlike rod-shaped bacteria, divides in three consecutive perpendicular division planes. The only known spatial regulator of division in *S*. *aurues* is the nucleoid occlusion protein Noc. In *Bacillus subtilis*, Noc has been shown to bind specific DNA sequences on the chromosome and block the assembly of the cell division apparatus over these sites. Because these binding sites are enriched in the origin-proximal portion of the chromosome and absent in the terminus region, Noc is thought to help coordinate cell division with chromosome segregation. Here, we report that *S*. *aureus* Noc protein not only plays a similar role in nucleoid occlusion, but also controls the initiation of DNA replication, thus providing an even tighter connection between cell division and chromosome biology than previously appreciated. This degree of economy in coordinating key cell biological processes may reflect the challenges posed by the small cell size and complicated division pattern of this spherical pathogen.

## Introduction

Binary fission in bacteria is carried out by a multiprotein complex called the divisome [1]. Assembly of this complex initiates with the formation of a ring-like structure at mid-cell called the Z-ring composed of the tubulin homolog FtsZ [2]. The Z-ring functions as a dynamic scaffold for the recruitment and assembly of the division machinery. In rod-shaped bacteria like *E*. *coli* and *B*. *subtilis*, midcell localization of the Z-ring is controlled by two negative regulatory systems: Min and nucleoid occlusion [1, 3]. The Min system prevents Z-ring assembly at the poles while nucleoid occlusion blocks its assembly over of the nucleoid; together they ensure that division occurs at mid-cell.

In *E*. *coli*, nucleoid occlusion is mediated by SlmA, a TetR family DNA binding protein [4]. SlmA directly inhibits FtsZ polymerization and this inhibition is stimulated by binding to specific DNA sequences [5–7]. In *B*. *subtilis*, nucleoid occlusion requires a ParB homolog called Noc [8]. The mechanism by which Noc inhibits Z-ring formation is less clear. Noc forms higher order nucleoprotein complexes [9] that associate with the cell membrane via an amino-terminal amphipathic helix and these complexes have been suggested to physically occlude the assembly of the division machinery [10]. Neither the Min system nor the nucleoid occlusion proteins are essential in *E*. *coli* or *B*. *subtilis* [4, 8, 11, 12]. However, defects in both regulatory systems results in a lethal division block [4, 8]. It was this synthetic lethal relationship that initially facilitated the identification of Noc and SlmA.

Importantly, the specific binding sites for SlmA and Noc are distributed throughout the origin-proximal portions of their respective chromosomes and are largely absent in the region surrounding the terminus [6, 9, 13]. During the chromosome replication-segregation cycle in both organisms, the origin regions are segregated toward the cell poles and the terminus is brought to mid-cell, where it remains for the majority of the cell cycle [14, 15]. As DNA replication nears completion, chromosome-bound nucleoid occlusion factors are depleted from mid-cell, relieving division inhibition at this site. Accordingly, SlmA and Noc are not only thought to function in division site selection, but also in coordinating cell division with chromosome replication and segregation [6, 9].

Unlike rod-shaped bacteria, where the same perpendicular plane is used for binary fission, cell division alternates between three consecutive perpendicular planes in the spherical bacterium *Staphylococcus aureus* [16]. The mechanisms governing division site selection in this major human pathogen are poorly understood. *S*. *aureus* does not encode the Min system, but does have a homolog of *B*. *subtilis* Noc (48% identity, 65% similarity). A previous study showed that *S*. *aureus* cells lacking Noc are impaired in division and on average are larger than wild-type [17]. Furthermore, Δ*noc* mutants displayed an elevated frequency of cells with multiple Z-rings and division events bisecting the chromosome. Thus, a role for Noc in the spatial regulation of division is conserved in *S*. *aureus*.

Even though *S*. *aureus* cells lack a Min system, Noc is not essential for viability [17]. Additional division regulators are thus likely to operate in this bacterium. By analogy with the Min^-^ Noc^-^ phenotype in rod-shaped organisms, we reasoned that defects in such regulators would result in a synthetically lethal or sick phenotype in combination with a *noc* deletion. To identify these factors we used transposon-sequencing (Tn-Seq) [18] to screen for genes that when inactivated result in a synthetically lethal with noc (Sln) phenotype. Two *sln* genes were identified in this screen, *comEB* and *rhomboid* (*rbd*). To further characterize the nature of the synthetic lethal defect of Δ*noc* Δ*rbd* and Δ*noc* Δ*comEB* mutants, suppressors that allowed growth of the double mutants were selected. Surprisingly, in both cases, suppressor mutations that mapped to the *dnaA* gene encoding the initiator of DNA replication were isolated, suggesting a role for Noc in controlling origin firing. Indeed, *S*. *aureus* cells lacking Noc were found to over-initiate replication, and the *dnaA* suppressor mutations ameliorated the over-replication phenotype and significantly reduced the severity of the division defects displayed by Δ*noc* cells. Reciprocally, we found that over-expression of DnaA enhanced the over-initiation and cell division phenotypes of cells lacking Noc. Altogether, our data reveal that *S*. *aureus* Noc coordinates division with chromosome replication and segregation by controlling replication initiation as well as preventing divisome formation over the origin-proximal region of the chromosome. This degree of economy in coordinating key cell biological processes may reflect the challenges posed by the smaller size and complicated division pattern of this spherical bacterium.

## Results

### A screen for synthetic lethal partners with *noc* identifies *rbd* and *comEB*

**Fig 1:**
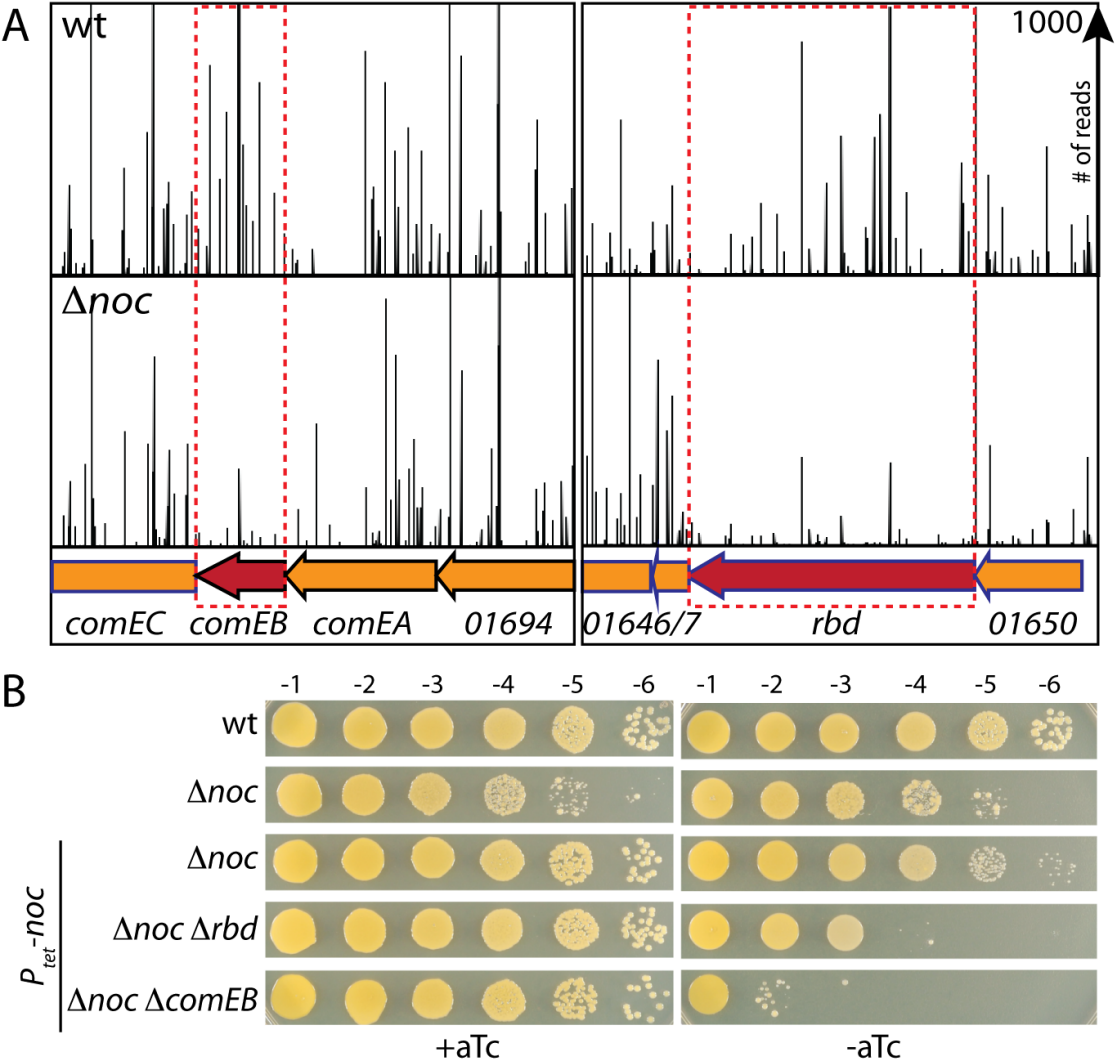
Identification of synthetic lethal partners of Δ*noc* in *S*. *aureus*. (**A**) Transposon insertion profiles for selected regions of the chromosome for both wild-type and Δ*noc* strains. The height of each line in the profile represents the number of sequencing reads corresponding to a transposon insertion at the indicated genome position. Transposon insertions in the *comEB* and *rbd* genes were significantly under-represented in the Δ*noc* mutant relative to wild-type. (**B**) Spot dilutions of wild-type HG003 (wt) and the indicated derivatives. Cells were grown overnight in the presence of aTc (100 ng/ml). The resulting cultures were normalized to an OD_600_ of 0.2, 10-fold serially diluted, and spotted (5 μl) onto agar medium with and without inducer as indicated. Plates were incubated overnight at 37°C and photographed.

To identify potential division site regulators, we screened for genes that become essential in the absence of *S*. *aureus* Noc (^*Sa*^Noc) using Transposon-sequencing (Tn-seq) [18]. A wild-type *S*. *aureus* RN4220 strain and a Δ*noc* derivative were mutagenized with a Mariner-based transposon [19]. The resulting libraries were separately pooled and the transposon-genomic DNA junctions of all insertions in the libraries were mapped by massively parallel DNA sequencing. For each library, we detected insertions in ~30% of the potential Mariner insertion sites at TA dinucleotides, and each insertion site was detected by an average of ~50 reads. Transposon insertions in several genes were statistically underrepresented in the Δ*noc* library compared to the wild-type library as assessed by the Mann-Whitney *U* test (**Fig 1A and S1 Table**). To validate the candidate synthetic lethal with Δ*noc* (*sln*) genes, we constructed a ^*Sa*^Noc depletion strain in which *noc* was expressed under the control of an anhydrotetracycline (aTc)-inducible promoter (*P*_*tet*_) in the *S*. *aureus* HG003 background. Transposon-insertion mutants in the candidate genes were obtained from the Nebraska Transposon Mutant Library [20] and transduced into the Noc-depletion strain grown in the presence of inducer and then tested for their ability to form colonies in its absence. Transposon insertions in *comEB* and *rbd* were the only mutants that resulted in a strong dependence on *noc* expression for viability (**S1 Table**). We therefore focused our analysis on these two factors. Insertion-deletion mutations in *comEB* and *rbd* were generated and the synthetic lethality upon depletion of Noc was confirmed (**Fig 1B**). The *rbd* gene encodes a member of the Rhomboid family of membrane-embedded serine proteases [21, 22]. ComEB is homologous to deoxycytidylate deaminase, an enzyme in one of the dTTP synthesis pathways [23].

### Inactivation of ComEB or Rbd enhances the division defects of Δ*noc* mutants

**Fig 2:**
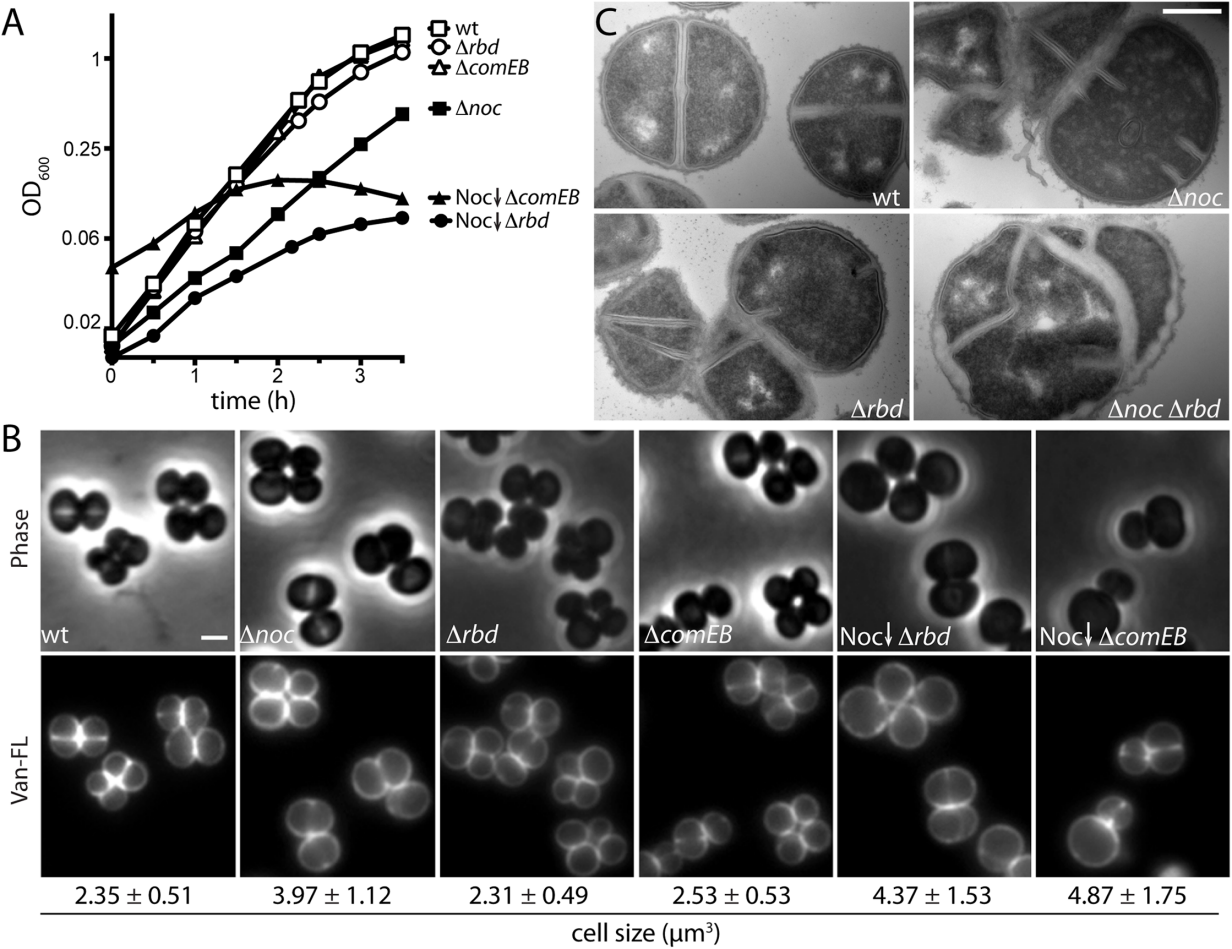
Characterization of the synthetic lethal with Δ*noc* phenotypes. (**A**) Growth curves of the indicated strains from Fig 1B. Overnight cultures grown in the presence of aTc (100 ng/ml) were washed to remove inducer and used to inoculate fresh medium without inducer to an OD_600_ of 0.01. The resulting cultures were grown at 37°C to an OD_600_ of 1, followed by a second round of dilution to an OD_600_ of 0.01 (or 0.04 for Noc depletion in the Δ*comEB* background). Growth during Noc depletion was then monitored by standard OD_600_ measurements. (**B**) Representative images of cells grown as described in A (for 5 mass doublings), except for the Noc depletion strains, which were examined after 13 mass doublings. Cultures were collected at OD_600_ ~ 0.4, washed and resuspended in phosphate buffered saline (1XPBS) prior to labeling with fluorescent vancomycin (Van-FL). Cells were visualized by phase contrast (Phase) and fluorescence (Van-FL) microscopy. Average cell size (μm^3^) ± standard deviation of each strain is indicated below each image (n>100). Scale bar indicates 1 μm. (**C**) Electron micrographs of the indicated strains. Overnight cultures of HG003 (wt) and mutant derivatives (Δ*noc*, Δ*rbd*, and Δ*noc* Δ*rbd*) were grown under permissive conditions (half-strength LB without NaCl (0.5XLB no NaCl) at 30°C) to an OD_600_ of 0.5. Cells were then washed, and diluted to an OD_600_ of 0.02 and grown under non-permissive conditions (LB with 0.5% NaCl medium at 37°C) for ~4 mass doublings. Cells were then harvested, processed, and visualized by electron microscopy as described in Methods. Scale bar indicates 500 nm. Larger fields of cells can be found in S1 Fig.

Next, we investigated the cytological phenotypes of the Noc^-^ ComEB^-^ and Noc^-^ Rbd^-^ mutants. Cells lacking Rbd or ComEB but with functional ^*Sa*^Noc grew at rates similar to wild-type (**Fig 2A**) and appeared similar in size and morphology by fluorescence microscopy (**Fig 2B**). By contrast and as expected, depletion of ^*Sa*^Noc in the mutant backgrounds resulted in severe growth defects (**Fig 2A**). Examination of these ^*Sa*^Noc-depleted cells by fluorescence microscopy after 6 hours of growth in the absence of inducer revealed an increase in cell size, indicative of a defect in cell division. Specifically, the average volume of cells lacking Noc was ~69% greater than wild-type, while cells depleted of ^*Sa*^Noc in the absence of Rbd or ComEB were even larger (~86% and ~107% greater than wild-type) (**Fig 2B**). To investigate the defects in cell division at higher resolution, we visualized stained thin-sections by transmission electron microscopy. Cells lacking ^*Sa*^Noc or Rbd alone displayed a low frequency (4–6%) of abnormal and incomplete septa that were not oriented orthogonal to each other (**Fig 2C and S1 Fig**). Such aberrant divisions were even more prevalent (~27%) in cells lacking both proteins, and the frequency of lysed cells also increased (~24%) (**Fig 2C, S1 Fig and S2 Table**). Collectively, these results indicate that the loss of ComEB or Rbd exacerbates the division defects of Δ*noc* cells, ultimately resulting in lysis.

### Mutations in *dnaA* suppress the synthetic lethal with Δ*noc* phenotypes

**Fig 3:**
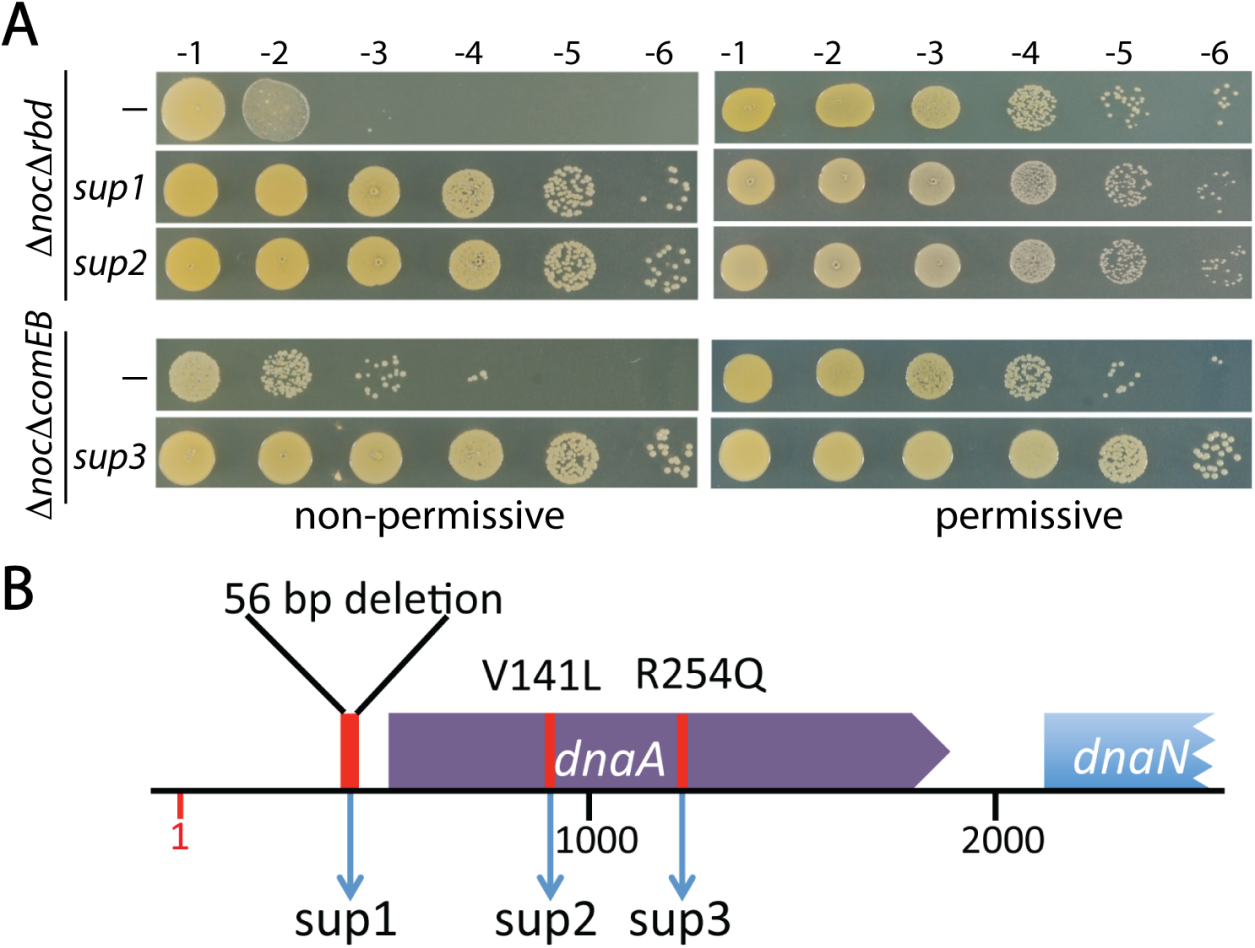
Suppressors of the Δ*noc* Δ*rbd* and Δ*noc* Δ*comEB* mutants map to the *dnaA* locus. (**A**) Spot dilutions of Δ*noc* Δ*rbd*, Δ*noc* Δ*comEB* and derivatives harboring the indicated suppressor mutation were grown under their respective permissive conditions overnight. They were then washed, serially diluted, and plated as described in Fig 1. (**B**). Schematic diagram of the *dnaA* locus indicating the location of each of the three suppressors isolated. Genome position 1 indicates the origin.

In an attempt to elucidate the potential roles of ComEB and Rbd in cell division we sought to identify suppressors of the observed synthetic lethal phenotypes. To do so, we first identified conditions that support growth of the Δ*comEB* or Δ*rbd* mutant upon depletion of ^*Sa*^Noc (**Fig 3A and S2 Fig**) (see Materials and Methods). The permissive plating conditions were then used to generate Δ*noc* Δ*comEB* and Δ*noc* Δ*rbd* double deletion mutants. To isolate suppressors, the double mutants were grown in liquid medium under permissive conditions and then plated on solid agar under restrictive conditions. Nine suppressors of each double mutant pair were mapped using whole genome sequencing and confirmed by Sanger sequencing (**S3 Table**). Two of the Δ*noc* Δ*rbd* suppressors and one of the Δ*noc* Δ*comEB* suppressors mapped to the *dnaA* gene encoding the replication initiator protein (**Fig 3B**) [24, 25]. One of the suppressors of Δ*noc* Δ*rbd* had a 56 bp deletion in the 5’ UTR of *dnaA* (*sup 1*), while another encoded a DnaA variant with a V141L substitution within the ATPase domain (Domain III) (*sup 2*). Similarly, a variant with an R254Q substitution in Domain III (*sup 3*) suppressed the Δ*noc* Δ*comEB* phenotype. Although we had hoped that the suppressors would shed light on a potential role for ComEB and Rbd in cell division regulation, the fact that mutations in *dnaA* suppressed both the Δ*noc* Δ*comEB* and Δ*noc* Δ*rbd* growth defects suggested that it was some aspect of the Noc^-^ phenotype that was most likely being corrected by the suppressors. These findings raised the intriguing possibility that ^*Sa*^Noc function is linked to DNA replication. Accordingly, we decided to focus the remainder of this study on this potential connection. The possible roles of ComEB and Rbd in cell division and the cause of the synthetic lethal with Δ*noc* phenotype will be the subject of a separate report (see Discussion).

### Cells lacking ^*Sa*^Noc over-replicate

**Fig 4:**
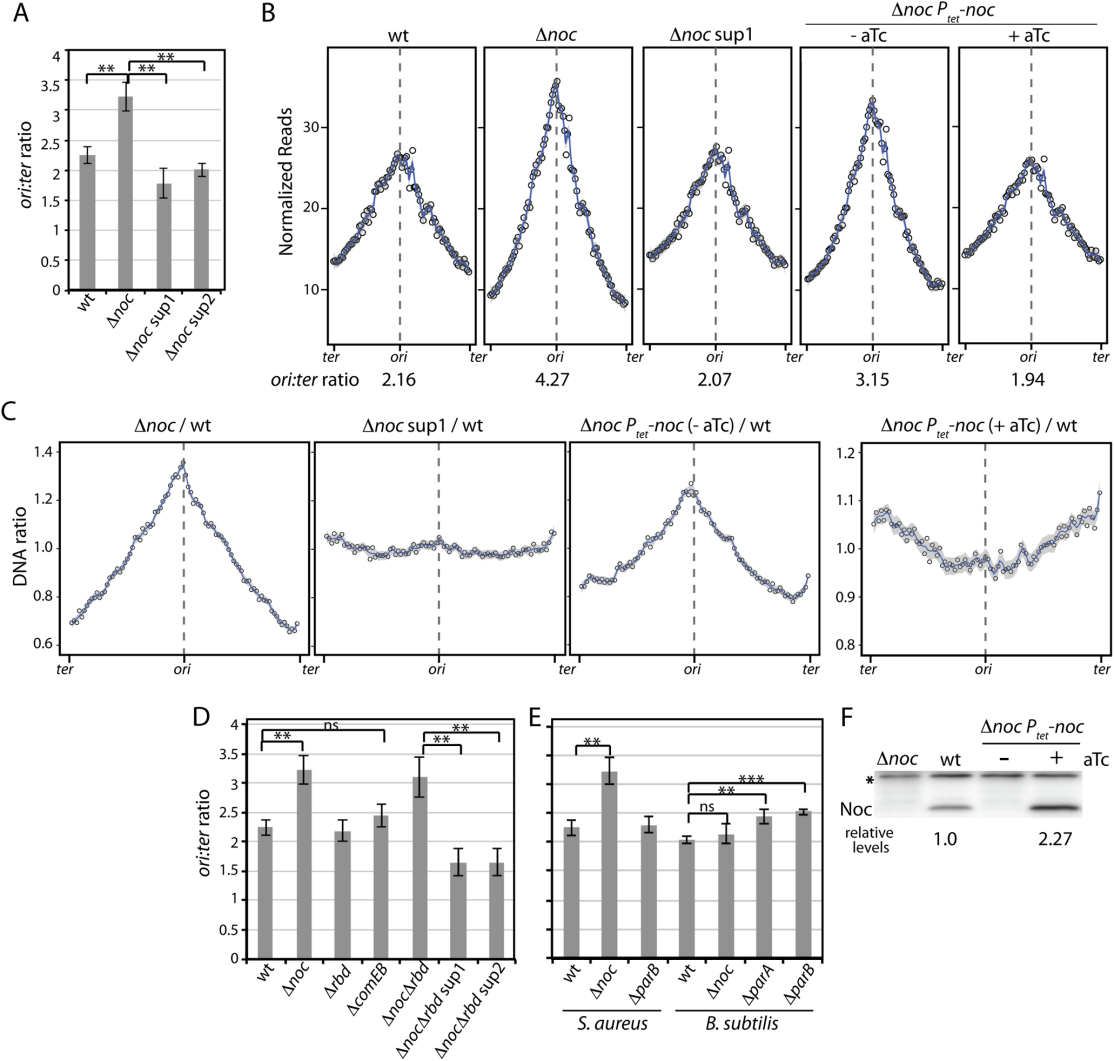
Over-replication phenotype of Δ*noc* mutants. Marker-frequency analysis of the indicated *S*. *aureus* and *B*. *subtilis* strains. (**A**) Cells of HG003 (wt) and its indicated derivatives were grown in LB with 0.5% NaCl medium at 37°C to OD_600_ ~ 0.4. Cells were then harvested and genomic DNA isolated. The *oriC*-to-*terminus* (*ori*:*ter*) ratio of each strain was then determined by quantitative PCR (qPCR) using primers specific for the relevant chromosomal regions. The results are shown as the average ± standard deviation (n = 3). The *p*-values of relevant pairs of samples were calculated and indicated on the bar graph. *p* > 0.05 (ns), *p* ≤ 0.05 (*), *p* ≤ 0.01 (**), *p* ≤ 0.001 (***). (**B**) Genome-wide DNA content of the indicated strains. Overnight cultures of *S*. *aureus* strain RN4220 (wt), Δ*noc*, Δ*noc* sup1, and Δ*noc* cells expressing *noc* under the control of the *P*_*tet*_ promoter were diluted to OD_600_ = 0.01 and grown in TSB medium without or with inducer (aTc) at 37°C. Genomic DNA was isolated after 5 mass doublings and analyzed by whole-genome sequencing. The total sequencing reads from each strain were normalized to 51 million and the data plotted in 30 kb bins (circles). Blue lines and grey area represent the smoothed conditional mean and 95% confidence band for the regression curve, respectively (spanS = 0.08). The data from one of two biological replicates are shown. The *ori*:*ter* ratios indicated below the plots were determined using the 30kb bins spanning the origin and terminus. The partial suppression of Δ*noc*, *P*_*tet*_-*noc* in the absence of aTc is likely due to leaky expression. (**C**) Ratio of genomic profiles. The genomic profiles in (B) were re-plotted as ratios relative to wild-type. (**D**) The *ori*:*ter* ratios of *S*. *aureus* HG003 (wt) and its indicated derivatives were determined by qPCR as described above. The Δ*noc* Δ*rbd* strain was first grown under permissive conditions (0.5X LB no NaCl, 30°C) to OD_600_ ~0.4, then back diluted into nonpermissive conditions (LB 0.5% NaCl medium, 37°C) at an OD_600_ ~0.04 and harvested at OD_600_ ~0.4. (**E**) Cells of *S*. *aureus* HG003 (wt) and its indicated derivatives were grown and the *ori*:*ter* ratios determined as described above. Similarly, *B*. *subtilis* strain PY79 (wt) and its indicated derivatives were grown in CH medium at 30°C. The *ori*:*ter* ratios were determined using similar primer sets and qPCR conditions as used for *S*. *aureus* (n = 3). The *ori*:*ter* ratios of *S*. *aureus* HG003 (wt) and Δ*noc* cells are replotted from (D) for direct comparison. (**F**). Immunoblot analysis of Noc protein levels in the indicated strains. Cell extracts were prepared from the same cultures used in (B). The relative Noc protein levels were determined by normalizing to the cross-reacting protein band (asterisk) and then to wt (n = 3, SD = 0.40).

DnaA is the replication initiation protein in most bacteria [24, 25]. It is an AAA+ ATPase that exists in both ATP- and ADP-bound forms. ATP-bound DnaA binds cooperatively to sequence elements (called DnaA boxes) in the replication origin, and catalyzes the unwinding of double stranded DNA. DNA unwinding leads to the ordered loading of helicase and the rest of the replication machinery followed by the initiation of bi-directional replication. To investigate whether cells lacking Noc have defects in the initiation of DNA replication we performed marker-frequency analysis using quantitative PCR (qPCR) and whole genome sequencing. This analysis revealed that the Δ*noc* mutant had a higher origin to terminus (*ori*:*ter*) ratio than wild-type cells and this increase could be suppressed by the *dnaA* suppressor mutations (**Fig 4A–4C**), raising the possibility that the absence of ^*Sa*^Noc causes over-initiation of DNA replication.

An increase in the *ori*:*ter* ratio can be caused by over-initiation of DNA replication or a reduction in replication elongation rates or fork stalling and/or collapse. The latter situation could be relevant in this case because cells lacking Noc have an increased frequency of chromosome guillotining [17], which would lead to fork stalling. To investigate the nature of the defect in the Δ*noc* mutant, we quantitatively assessed DNA content relative to cell volume as a proxy for cell mass in single cells [26, 27]. Fixed permeabilized cells were stained with propidium iodide and analyzed by phase contrast and fluorescence microscopy. Consistent with the idea that cells lacking ^*Sa*^Noc over-initiate, the Δ*noc* mutant had a higher DNA content relative to cell volume compared to wild-type and the *dnaA* (*sup1*) mutation restored this ratio to wild-type levels (**S3 Fig**). Further support for a role for Noc in controlling replication initiation comes from an experiment in which we attempted to correct the increase in the *ori*:*ter* ratio by expressing *noc* in trans from an aTc-inducible promoter. As anticipated, modest (~2.3-fold) over-production of Noc in the Δ*noc* mutant (**Fig 4F**) led to a reduction in the *ori*:*ter* ratio (**Fig 4B**). However, comparison of the genomic profile relative to wild-type (**Fig 4C and S3 Fig**) revealed that the *ori*:*ter* ratio was even lower than wild-type, consistent with the idea that Noc acts as a negative regulator of replication initiation. Finally, to address whether the increase in the *ori*:*ter* ratio in the Δ*noc* mutant resulted from fork stalling due to division on top of the chromosomes, we performed genome-wide marker frequency analysis on wild-type and Δ*noc* strains before and after inhibition of cell division using the FtsZ inhibitor PC190723 [28]. We carried out whole genome sequencing of wild-type and the Δ*noc* mutant prior to the addition of the drug and approximately 2 and 4 mass doublings after its addition. Despite the strong block to cell division, the increase in the *ori*:*ter* ratio in the Δ*noc* mutant persisted (**S4A Fig**). Importantly, we found that DNA content increased after drug addition (**S4B Fig and S5 Fig**), indicating that the division-inhibited cells continued DNA replication. Thus, chromosome guillotining cannot account for the increase in the *ori*:*ter* ratio in the Δ*noc* mutant. Altogether, these experiments are most consistent with a model in which ^*Sa*^Noc functions to limit replication initiation and in its absence cells over-initiate.

Marker-frequency analysis in cells lacking Rbd or ComEB revealed that these mutants were not impaired in replication initiation (**Fig 4D**). Furthermore, the *ori*:*ter* ratio in the Δ*noc* Δ*rbd* double mutant was similar to the Δ*noc* single mutant (**Fig 4D**), indicating that the Rbd^-^ defect did not exacerbate the over replication phenotype of Noc^-^ cells.

Because ^*Sa*^Noc shares 48% identity with its *B*. *subtilis* counterpart (^*Bs*^Noc), we wondered whether *B*. *subtilis* cells lacking Noc also over-initiate. Marker-frequency analysis revealed that the *B*. *subtilis* Δ*noc* mutant had an *ori*:*ter* ratio similar to wild-type (**Fig 4E**). As a control, we examined the *ori*:*ter* ratios of *B*. *subtilis* cells lacking the ParB homolog Spo0J and the ParA homolog Soj. Previous studies indicate that Soj (ParA) functions as both an activator and inhibitor of DnaA, while Spo0J (ParB) promotes the inhibitory activity of Soj [29, 30]. As reported previously, both mutants had over-initiation phenotypes (**Fig 4E**), but neither was as pronounced as in *S*. *aureus* Δ*noc* cells. In addition to ^*Sa*^Noc, *S*. *aureus* cells encode a second ParB-like protein (^*Sa*^ParB) that is 47% identical to Spo0J (^*Bs*^ParB), although they lack a ParA counterpart. A mutant lacking ^*Sa*^ParB had an *ori*:*ter* ratio similar to wild-type cells (**Fig 4E**), indicating that among the ParB related proteins in *S*. *aureus*, a role in replication control is restricted to ^*Sa*^Noc.

### Chromosomal distribution of ^*Sa*^Noc binding sites

**Fig 5:**
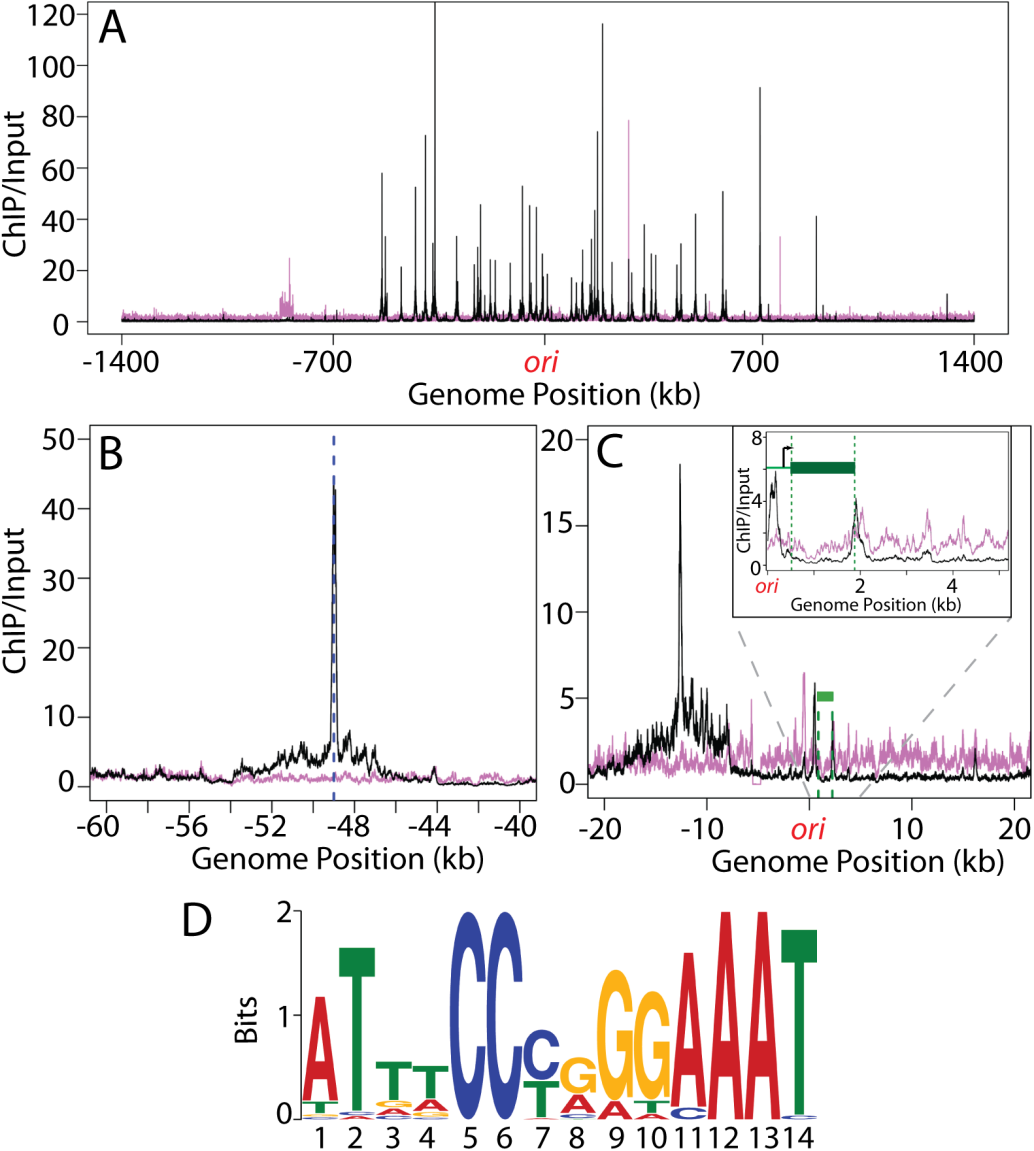
Identification of ^*Sa*^Noc binding sites. (**A**) Shown are the ChIP-Seq profiles for HG003 (wt) (black) and a Δ*noc* derivative (magenta). The profiles were generated by normalizing the reads from ChIP-Seq samples (using anti-^*Bs*^Noc antibodies) to those from input genomic DNA. The origin (*ori*) is at position 1 with positions on the left and right chromosome arms indicated as negative and positive numbers, respectively. (**B**) Close-up view of a region bound by Noc with the peak indicated by a dotted blue line. Note the extended spread of the peak base compared to the background signal from the Δ*noc* sample. (**C**) Close-up view of the origin region including a minor Noc binding site adjacent to the *dnaA* gene (green bar). The location and direction of the *dnaA* transcription start site are indicated by the black arrow in the inset. (**D**) Sequence logo of the ^*Sa*^Noc consensus binding site generated using the 41 major enrichment peaks (S7 Table) identified in the ChIP-Seq analysis.

In *B*. *subtilis*, ^*Bs*^Noc binds multiple sites throughout the chromosome that are enriched around the origin-proximal two-thirds of the chromosome and largely absent from the replication terminus region [9]. This distribution of binding sites is thought to ensure that Z-ring formation is coordinated with chromosome segregation [9]. Because our data suggest that in *S*. *aureus* Noc regulates replication initiation, we wondered whether it might have a distribution of DNA binding sites distinct from ^*Bs*^Noc and different from that predicted based on mapping the sequence of the ^*Bs*^Noc binding sites on the *S*. *aureus* chromosome [31]. To investigate this we determined the *S*. *aureus* Noc binding sites (NBS) by chromatin-immunoprecipitation coupled to Illumina sequencing (ChIP-seq). We used antibodies raised against ^*Bs*^Noc, which cross-reacted with the *S*. *aureus* homolog. A total of 41 chromosomal loci were identified that co-precipitated with ^*Sa*^Noc from wild-type cells compared to the Δ*noc* mutant (**Fig 5A and S7 Table**). Importantly, all but one of these loci were located in the origin-proximal half of the chromosome (between -700kb and +700kb), resulting in a distribution of binding sites that resembles the *B*. *subtilis* pattern [31]. Furthermore, as is the case in *B*. *subtilis* [9], ^*Sa*^Noc binding spanned a region of 8–10 kb surrounding the peak of enrichment (**Fig 5B and 5C**), suggesting that *S*. *aureus* Noc forms large nucleoprotein complexes at its binding sites. Finally, using motif discovery algorithms from the MEME suite [32], the consensus Noc binding site was determined, the core of which was found to be identical to the *B*. *subtilis* NBS consensus sequence [31] (**Fig 5D**). The similar distribution of Noc binding sites, the identical consensus binding sequence, and the shared ability of the Noc homologs to spread from these sites are all consistent with ^*Sa*^Noc functioning as a nucleoid occlusion factor in addition to its role in replication control identified here.

### *B*. *subtilis noc* and *S*. *aureus noc* do not cross-complement

**Fig 6:**
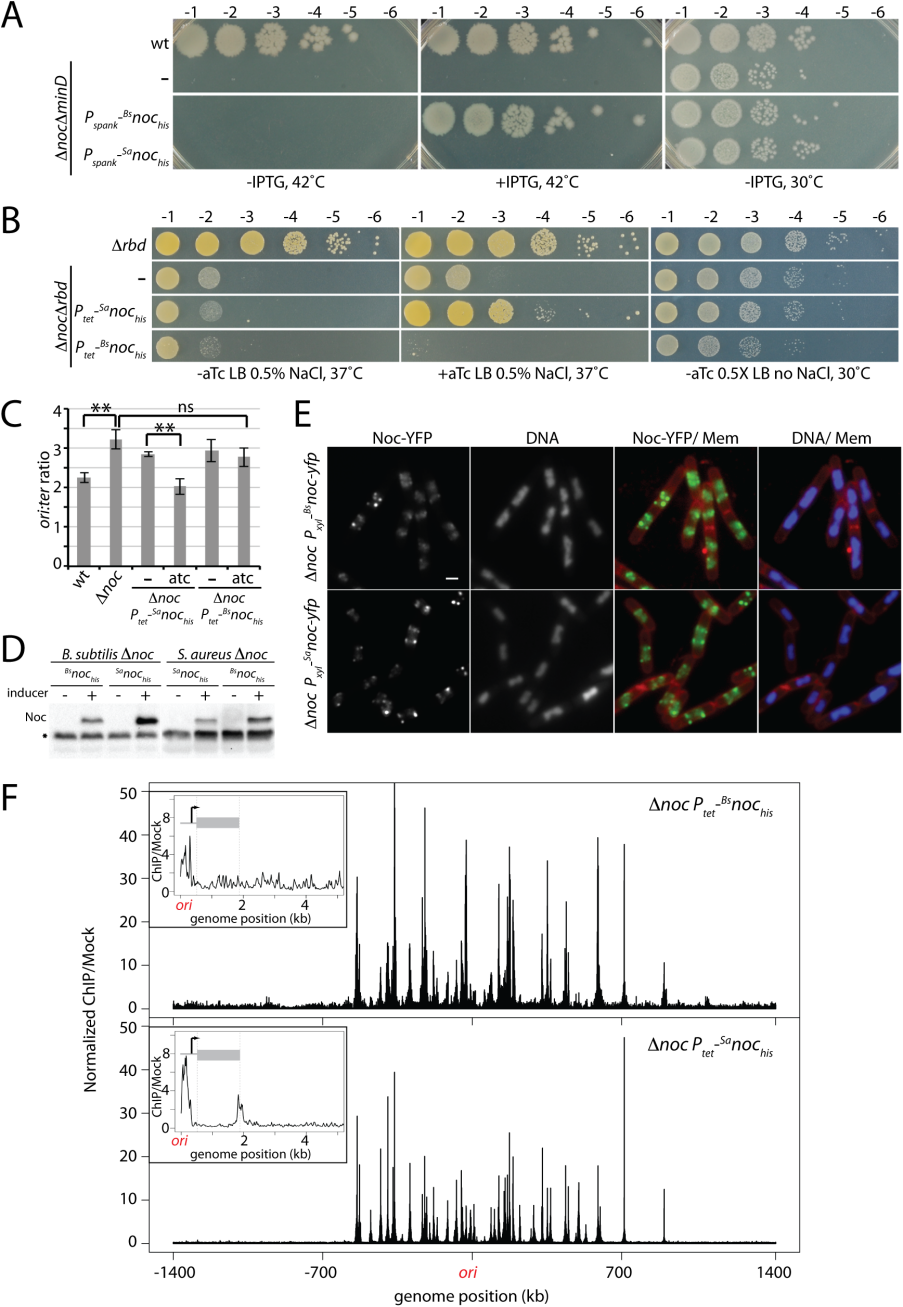
^*Sa*^Noc and ^*Bs*^Noc are not functionally interchangeable. (**A**) Spot-dilutions of *B*. *subtilis* strain PY79 (wt) and the indicated derivatives harboring *his*-tagged *noc* fusions from either *B*. *subtilis* (^*Bs*^*noc*_*his*_) or *S*. *aureus* (^*Sa*^*noc*_*his*_) expressed from the IPTG-inducible *P*_*spank*_ promoter. Cells were grown overnight and the resulting cultures were normalized to an OD_600_ of 0.2 and serially diluted. 5 μl of each dilution were spotted onto LB agar with or without inducer and the plates were incubated overnight at the indicated temperatures. (**B**) Spot-dilutions of *S*. *aureus* HG003 (wt) and its indicated derivatives. Cells were grown, serially diluted, and plated as described in (A). In these strains the *noc* fusions were expressed from the aTc-inducible *P*_*tet*_ promoter. (**C**) Marker-frequency analysis of the indicated *S*. *aureus* strains was performed as in Fig 4. The results shown are the average ± standard deviation (n = 3). Results for wt and Δ*noc* cells are replotted from Fig 4. *p* > 0.05 (ns), *p* ≤ 0.01 (**). (**D**) Immunoblot analysis of Noc protein levels in the indicated strains. Cell extracts representing equivalent culture OD_600_ were prepared from *B*. *subtilis* and *S*. *aureus* strains grown in LB or TSB, respectively at 37°C with or without inducer. Proteins were separated by SDS-PAGE, transferred to PVDF membrane, and probed with an anti-6xHis antibody. A cross-reacting protein (asterisk) serves as a loading control. (**E**) ^*Sa*^Noc-YFP and ^*Bs*^Noc-YFP localize to the peripheral membranes above the nucleoid in *B*. *subtilis*. Representative images of Δ*noc B*. *subtilis* cells expressing ^*Bs*^*noc-yfp* or ^*Sa*^*noc-yfp* under the control of the *P*_*xyl*_ promoter. Cells were induced at OD_600_ = 0.01 with 0.5% xylose and analyzed by fluorescence microscopy at OD_600_ = 0.25. Noc-YFP, DAPI-stained DNA and merged images with Nile Red-stained membranes (Mem) are shown. Scale bar indicates 1 μm. Larger fields can be found in S6 Fig. (**F**) ^*Bs*^Noc_*his*_ and ^*Sa*^Noc_*his*_ have largely overlapping binding sites in *S*. *aureus*. ChIP-seq using the anti-6xHis antibodies was performed on *S*. *aureus* Δ*noc* cells expressing ^*Bs*^*noc*_*his*_ or ^*Sa*^*noc*_*his*_ under the control of the *P*_*tet*_ promoter. Wild-type and Δ*noc* cells lacking a *his*-tagged *noc* fusion were used as negative controls. Data presented were first normalized to the total number of reads then relative to the input for each strain and finally relative to that of the relevant control sample (wild-type cells for ^*Sa*^*noc*_*his*_ and Δ*noc* cells for ^*Bs*^*noc*_*his*_). The data are plotted in 1 kb bins. The higher background and higher peaks in the ^*Bs*^Noc_*his*_ ChIP-seq profile are due to fewer and sparser sequencing reads in this sample and the normalization process used. Genome positions are labeled as in Fig 5A. Insets show a close-up view of the origin region including a minor Noc binding site adjacent to the *dnaA* gene (gray bar). The data are plotted in 10 bp bins.

Since the Noc proteins from *B*. *subtilis* and *S*. *aureus* are 48% identical and they have the same consensus binding site, we investigated whether they could substitute for each other in vivo. *B*. *subtilis* cells lacking the Min system and Noc are viable at 30°C but fail to grow at 42°C [8]. Accordingly, we generated *his6* fusions to both *B*. *subtilis* and *S*. *aureus noc* under the control of an IPTG-inducible promoter (*P*_*spank*_) and introduced these into a Δ*minD* Δ*noc* double mutant under permissive conditions. Surprisingly, only *B*. *subtilis noc* was able to complement the growth defect of the double mutant at 42°C (**Fig 6A**). Similarly, we took advantage of the synthetic lethal phenotype of Δ*noc* Δ*rbd* in *S*. *aureus*. We introduced the same *his6* fusions under the control of an aTc-inducible (*P*_*tet*_) promoter into the double mutant at the L54a phage attachment site. Again, only the native *noc* allele supported growth under the non-permissive condition (**Fig 6B**). Furthermore, not only was *B*. *subtilis noc* incapable of supporting growth of the *S*. *aureus* Δ*noc* Δ*rbd* mutant, it was unable to correct the over-initiation defect of the *S*. *aureus* Δ*noc* mutant (**Fig 6C**). Importantly, immunoblot analysis using anti-6xHis antibodies indicated that in both complementation tests, the heterologous Noc proteins were produced at comparable or even higher levels than the native proteins (**Fig 6D**). Furthermore, as has been observed previously with ^*Bs*^Noc [9], a YFP fusion to ^*Sa*^Noc expressed in *B*. *subtilis* localized in foci along the cell periphery indicative of it associating with membranes surrounding the nucleoid in addition to binding DNA (**Fig 6E and S6 Fig**). Finally, ChIP-seq experiments using anti-6xHis antibodies revealed that both His6-Noc fusions bound to virtually all the same chromosomal sites in *B*. *subtilis* and *S*. *aureus* (**Fig 6F, S7 Fig, S8 Fig and S7 Table**). Thus, merely binding to the NBS sites throughout the chromosome is not sufficient for Noc function, suggesting that the Noc orthologs require species-specific contacts with replication and/or division factors and that these interaction interfaces are not conserved between the two proteins (See Discussion).

### Evidence that *S*. *aureus* Noc acts post-translationally to restrain replication initiation

**Fig 7:**
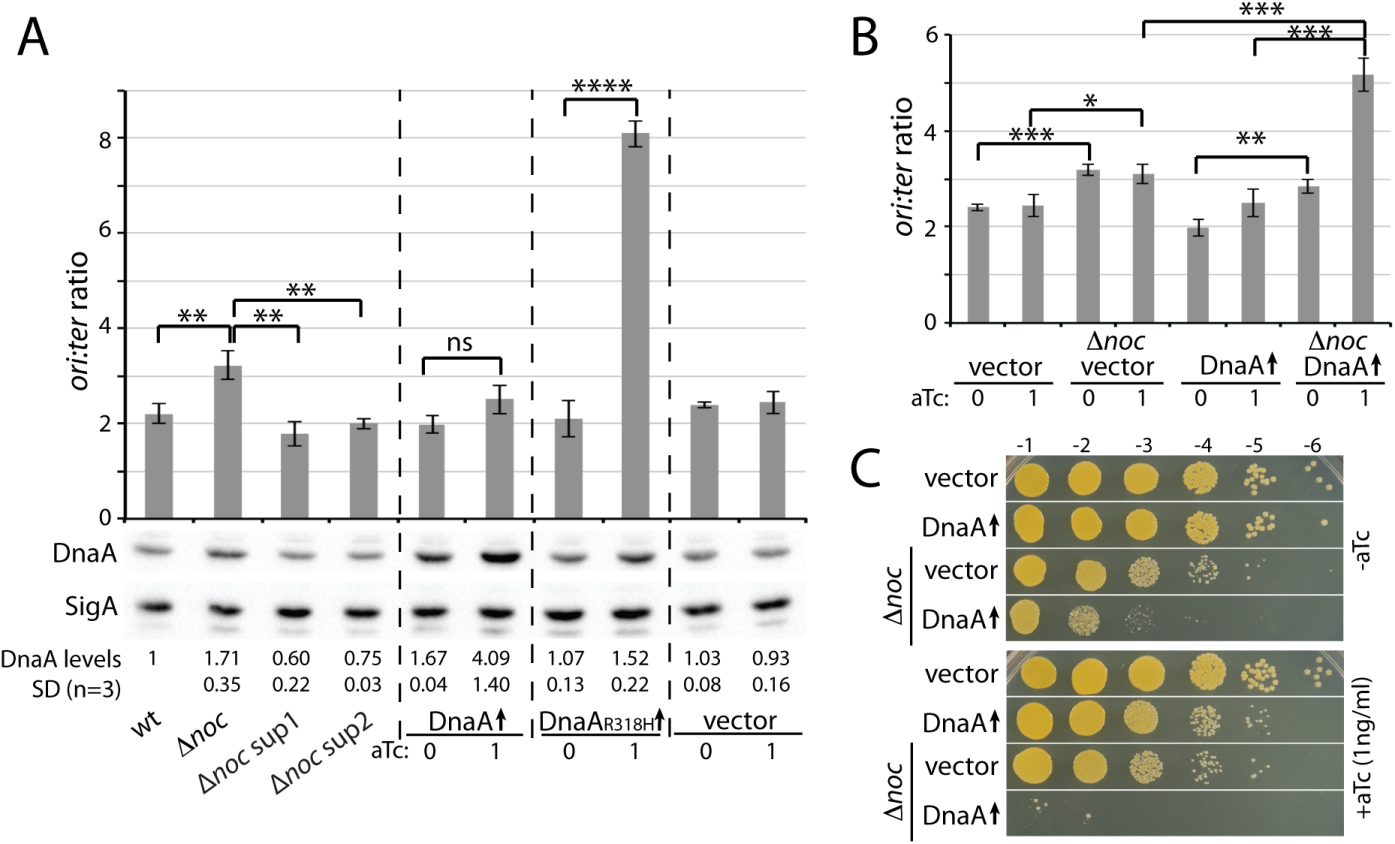
^*Sa*^Noc and DnaA have opposing activities. (**A**). Marker-frequency analysis of the indicated strains. Cultures were grown to OD_600_ = 0.4 and then processed to determine *ori*:*ter* ratios by qPCR (top panel) as in Fig 4 or to quantify DnaA protein levels by immunoblot (bottom panel). In induced cultures, 1 ng/ml aTc was added at OD_600_ = 0.05. *oriC*-to-*terminus* (*ori*:*ter*) ratios are shown as the average ± standard deviation (n = 3). *p* > 0.05 (ns), *p* ≤ 0.05 (*), *p* ≤ 0.01 (**), *p* ≤ 0.001 (***), *p* ≤ 0.0001 (****). To quantify DnaA levels, the immunoblot signal was normalized to SigA and reported relative to the wild-type strain that was designated as having a signal of 1. Standard deviation was indicated as SD (n = 3). (**B**). Marker-frequency analysis of the indicated strains. Overnight cultures of Δ*noc* cells carrying either an empty vector (vector) or a plasmid containing *dnaA* under *P*_*tet*_ control were diluted to an OD_600_ = 0.01, then grown to an OD_600_ of 0.05 at which time the indicated amount of inducer was added. Cells were then harvested at OD_600_ = 0.4 and the *ori*:*ter* ratios were determined as in Fig 4. Results shown are the average ± standard deviation (n = 3). Results for wt cells carrying empty vector or the *dnaA*-containing plasmid are replotted from (A). (**C**). Spot-dilutions of wild-type *S*. *aureus* HG003 and its Δ*noc* derivative carrying plasmids as in (B). Cultures were diluted to an OD_600_ of 0.15, serially diluted, and plated on the indicated medium as in Fig 1. Plates were incubated overnight at 37°C and photographed.

The ^*Sa*^Noc enrichment profile from the ChIP-seq experiment contained a small peak of enrichment within the promoter region of the *dnaA* gene (**Figs 5C and 6F**). Although ^*Bs*^Noc expressed in *S*. *aureus* contained a similar enrichment peak, we wondered whether ^*Sa*^Noc functions in replication control by regulating *dnaA* expression. Accordingly, we compared the relative levels of DnaA and the house-keeping sigma factor SigA by immunoblot analysis using anti-DnaA and anti-SigA antibodies. Both antibodies were raised against the *B*. *subtilis* proteins but cross-reacted with their *S*. *aureus* orthologs. Intriguingly, we detected a 1.7-fold increase in DnaA levels in cells lacking ^*Sa*^Noc compared to wild-type (**Fig 7A**). Furthermore, the *dnaA* suppressor mutations reduced the levels of DnaA in the Δ*noc* mutant to a level slightly below wild-type. To directly test whether the over-initiation phenotype in cells lacking ^*Sa*^Noc was due to this increase in DnaA levels, we compared the *ori*:*ter* ratio in a strain harboring an aTc-inducible promoter fusion to *dnaA*. Consistent with previous results, we did not observe a correlation between mild DnaA overproduction and over-initiation of DNA replication in *S*. *aureus* [33]. In the absence of inducer, the leaky expression from this plasmid-borne *dnaA* allele resulted in DnaA levels comparable to the Δ*noc* mutant (**Fig 7A**). However and importantly, the *ori*:*ter* ratio in this strain was similar to wild-type and a matched empty vector control strain. Furthermore, even higher levels of DnaA in the presence of inducer did not cause over-initiation compared to the control strain (**Fig 7A**). As a positive control for these experiments we used a strain harboring the same aTc-inducible promoter fused to an ATPase defective allele of *dnaA* (*dnaA*_R318H_). As reported previously [33], induction of DnaA_R318H_ caused a severe growth defect and led to dramatic over-initiation of replication (**Fig 7A**). We conclude that Noc directly or indirectly affects DnaA levels but these changes are not the major determinant of the observed effects on DNA replication.

If, as our data suggest, Noc negatively regulates replication initiation at the post-translational level, then overproduction of DnaA in a Δ*noc* mutant should exacerbate the over-initiation phenotype. As can been seen in **Fig 7B**, overexpression of *dnaA* in cells lacking Noc dramatically increased replication initiation with an *ori*:*ter* ratio approaching the levels reached upon DnaA_R318H_ production in wild-type cells. Furthermore, similar to what has been reported previously for the ATPase deficient DnaA variant [33], this level of over-replication led to a dramatic growth defect (**Fig 7C**) and a significant increase in cell size (**S9 Fig**) (see below). Altogether these data argue that Noc exerts its control on replication initiation through a post-translational mechanism.

### *dnaA* suppressors partially suppress the cell division defect in Δ*noc* mutants

**Fig 8:**
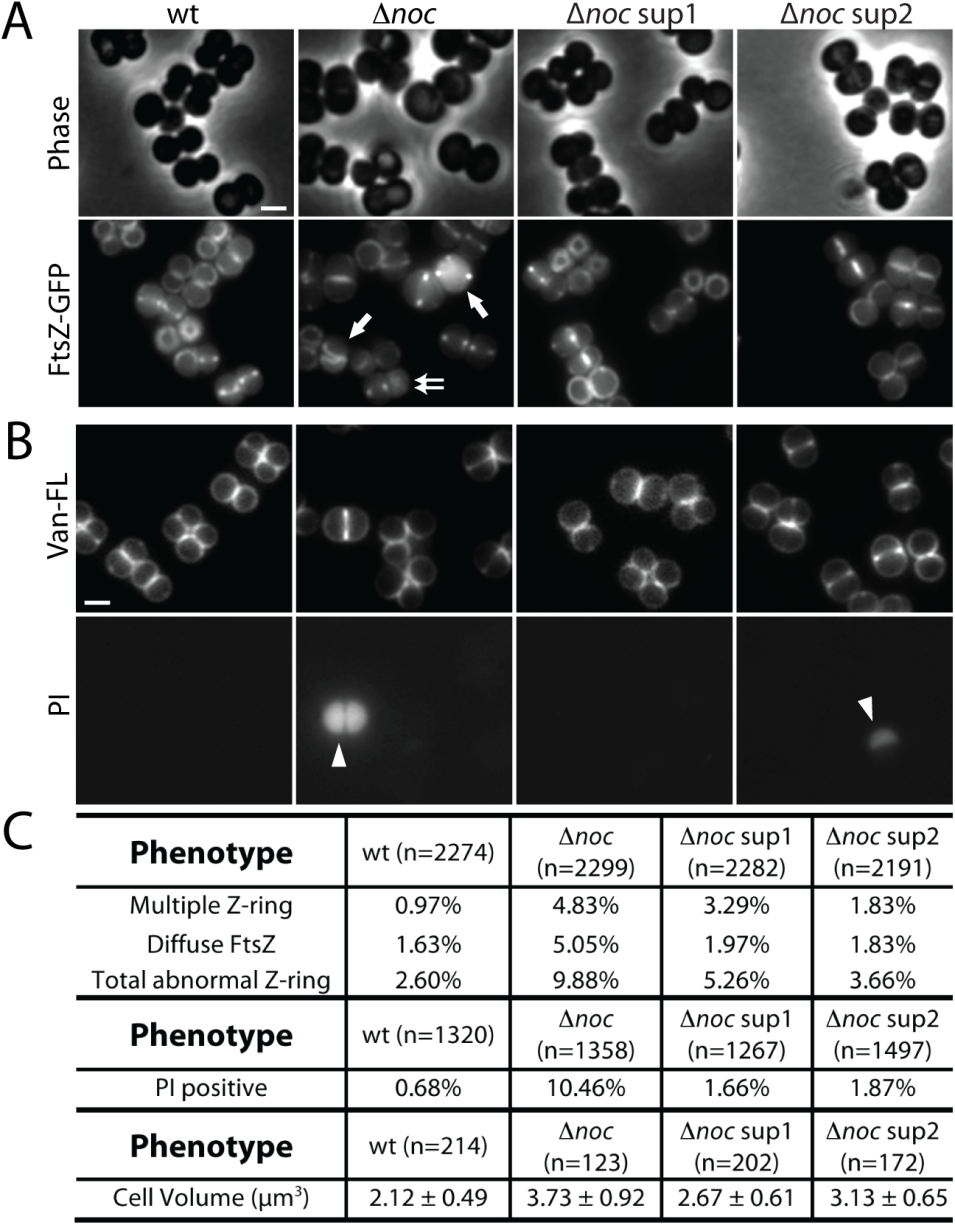
The cell division phenotypes in cells lacking ^*Sa*^Noc are partially due to over-initiation of replication. (**A**) Representative images of HG003 (wt) and derivatives harboring the plasmid pLOW-*ftsZ-gfp*. Cells were grown to an OD_600_ ~ 0.01 and FtsZ-GFP production was induced with 10 μM IPTG. When cultures reached an OD_600_ ~ 0.4, cells were resuspended in 1XPBS and visualized using phase contrast and fluorescence microscopy. Scale bar indicates 1 μm. (**B**) The same set of strains, but without the pLOW-*ftsZ-gfp* plasmid, were grown to an OD_600_ = 0.4, resuspended in 1XPBS, stained with BODIPY-Vancomycin (Van-FL) and propidium iodide (PI), and visualized by fluorescence microscopy. Examples of multiple FtsZ-rings (single arrow), diffuse FtsZ signal (double arrow), and PI-positive cells (arrowhead) are indicated. (**C**) Quantification of the FtsZ-ring formation phenotypes, frequency of PI staining, and cell size in the indicated strains.

Previous studies indicate that *S*. *aureus* cells lacking ^*Sa*^Noc display division defects including oblique FtsZ-rings, an increase in cell size, and the production of anucleate cells [17] (**Fig 2B and 2C**). Here, we have shown that the Δ*noc* mutant also over-initiates DNA replication. To investigate the contribution of over-initiation to the division defects in cells lacking ^*Sa*^Noc, we took advantage of the suppressor mutations isolated in *dnaA*. We analyzed cell size and FtsZ localization by fluorescence microscopy in the Δ*noc* mutant and in the Δ*noc* mutant harboring *dnaA*(*sup1*) or *dnaA*(*sup2*). Consistent with our observation that over-initiation leads to an increase in cell size (**S9 Fig**), both *dnaA* mutations partially suppressed the large cell phenotype of the Δ*noc* mutant (**Fig 8C and S10 Fig**). To monitor FtsZ localization we used an FtsZ-GFP fusion as a merodiploid [34] in the same set of strains. Interestingly, the suppressors reduced the percentage of cells with abnormal Z-rings (**Fig 8A and 8C**). In particular, we observed a reduction in oblique Z-rings and in the percentage of cells with diffuse FtsZ-GFP fluorescence. Furthermore, we also found that ~10% of cells lacking ^*Sa*^Noc exhibited extremely strong DAPI staining (**S10 Fig**). We suspected this phenotype was due to increased cell envelope permeability in the mutant, and staining cells with the membrane-impermeable dye propidium iodide (PI) confirmed this (**Fig 8B**). Importantly, the *dnaA* suppressors alleviated this hyper-staining/membrane permeability phenotype such that it was comparable to wild-type cells (**Fig 8B and 8C**). Collectively, these data suggest that the cell division problems experienced by *S*. *aureus* cells lacking ^*Sa*^Noc are partially due to the over-initiation of DNA replication.

## Discussion

The initial goal of this study was to identify additional regulators of division site placement in *S*. *aureus* by screening for synthetic lethal partners of the nucleoid occlusion factor ^*Sa*^Noc. The Tn-Seq analysis successfully identified Rbd and ComEB, annotated as a rhomboid-family protease and a deoxycytidylate deaminase, respectively as being essential upon ^*Sa*^Noc inactivation. Although we intended to use the synthetic lethal phenotype of Noc^-^ Rbd^-^ or Noc^-^ ComEB^-^ mutants to learn more about Rbd and ComEB function, our genetic analysis led to the surprising discovery that ^*Sa*^Noc plays a role in controlling the initiation of DNA replication as well as functioning as a nucleoid occlusion factor. We therefore shifted our focus to studying this newfound function for Noc instead of further investigating the activities of Rbd and ComEB. Consequently, the underlying cause(s) of the synthetic lethal phenotypes we observe for Δ*noc* Δ*rbd* or Δ*noc* Δ*comEB* strains remain unclear.

Defects in Rbd or ComEB were not found to increase the *ori*:*ter* ratio of wild-type or Δ*noc* cells, indicating that the synthetic lethality in combination with Δ*noc* is not due to additional problems constraining replication initiation. However, the possible role of ComEB in nucleotide metabolism may exacerbate the phenotype of Δ*noc* cells by limiting the availability of nucleotides needed to fuel the elevated level of DNA replication. Potential explanations for the Δ*noc* Δ*rbd* phenotype are more numerous. Aggravation of the replication, division, or both phenotypes of Noc^-^ cells is possible upon *rbd* inactivation, and in either case the added problems may be due to a failure in substrate processing by Rbd rather than a direct role in these processes for Rbd itself. Further studies of Rbd function aided by the strong growth phenotype of Δ*noc* Δ*rbd* cells identified here should shed light on the physiological role of rhomboid-like proteases in bacteria, which is poorly understood in general.

### Control of DNA replication by ^*Sa*^Noc

The elevated *ori*:*ter* ratio observed in Δ*noc* cells and its suppression by mutations in *dnaA* are consistent with a model in which ^*Sa*^Noc controls replication initiation. Further support for this idea comes from our findings that cells lacking Noc have elevated DNA content relative to cell volume and cells with increased Noc protein levels have a reduction in the *ori*:*ter* ratio relative to wild-type. Finally, the synthetic growth phenotype and increased *ori*:*ter* ratio in Δ*noc* mutant cells that over-expresses DnaA also argue for a role in replication initiation. However, because of the limited tools available to study replication in *S*. *aureus*, we cannot rule out the possibility that Noc also functions in replication elongation [35]. How ^*Sa*^Noc controls replication initiation remains unclear. Our finding that overexpression of *dnaA* does not lead to increased replication initiation unless Noc is inactivated indicates that ^*Sa*^Noc is unlikely to be regulating origin firing by affecting *dnaA* expression. Furthermore, the failure of ^*Bs*^Noc to functionally substitute for ^*Sa*^Noc even though it binds to almost all the same chromosomal sites as ^*Sa*^Noc suggests that site-specific DNA-binding activity alone is unlikely to account for replication control. However, we note that our ChIP-seq analysis identified two small enrichment peaks for ^*Sa*^Noc surrounding the *dnaA* gene while expression of ^*Bs*^Noc in *S*. *aureus* only had one of these small peaks (**Fig 6F**). Intriguingly, the predicted DnaA binding sites required for origin unwinding are located in these two regions [33, 36]. Accordingly, it is formally possible that ^*Sa*^Noc binding to these sites occludes DnaA and inhibits origin firing. Alternatively, this second enrichment peak could be reporting on an interaction between Noc and DnaA bound to the origin (see below). Because this intergenic region is critical for origin firing [37], we were unable to investigate whether or not Noc binding at this position is important for its role in replication control.

We hypothesize that ^*Sa*^Noc recognizes a specific molecular target to mediate its effect on replication initiation and that the interaction interface with this target is not conserved in ^*Bs*^Noc. DnaA is an obvious candidate as a direct regulatory target of ^*Sa*^Noc, but so far we have not been able to detect a ^*Sa*^Noc-DnaA interaction. Further work will therefore be required to determine the precise mechanism by which ^*Sa*^Noc controls initiation. Nevertheless, the discovery that ^*Sa*^Noc controls replication initiation in addition to its role in nucleoid occlusion provides a striking example of a single protein that can coordinate cell division and DNA replication by affecting both major cell-cycle processes.

It is noteworthy that mutations in *dnaC* encoding the replicative helicase were also identified as suppressors of Δ*noc* Δ*rbd* and Δ*noc* Δ*comEB* (**S3 Table**). Furthermore, at least one of these mutations (*dnaC*_*A352V*_) was able to suppress the over-initiation phenotype of the Δ*noc* Δ*rbd* mutant (**S11 Fig**). Since DnaC is required for both replication initiation and elongation [38] the molecular basis of suppression remains unclear. In the context of the model that Noc negatively regulates initiation, we hypothesize that these DnaC variants are specifically impaired in initiation function, however, further analysis will be required to establish the defects in these mutants.

### Mechanism of cell division regulation by Noc proteins

The mechanism by which Noc proteins block cell division over the nucleoid has remained unclear for many years. Attempts at identifying a specific component of the divisome targeted by ^*Bs*^Noc have been unsuccessful [10]. However, this protein was recently shown to have an amino-terminal amphipathic helix that recruits DNA-bound Noc complexes to the membrane [10]. Based on this result, it has been proposed that, rather than interacting with a specific component of the division apparatus, ^*Bs*^Noc nucleoprotein complexes at the membrane physically occlude the assembly of the division machinery in areas occupied by the origin-proximal portion of the chromosome [10]. If true, the only activities of Noc required for its control of cell division are its association with Noc-binding sites on the chromosome and their recruitment to the membrane. Given that ^*Sa*^Noc binds to the same sites as ^*Bs*^Noc, forms higher order nucleoprotein complexes and, like ^*Bs*^Noc, appears to associate with the membrane in addition to DNA, the physical occlusion model would predict that ^*Sa*^Noc should be able to substitute for ^*Bs*^Noc. However, we find that ^*Sa*^Noc fails to support growth of Min^-^ Noc^-^ *B*. *subtilis* cells under non-permissive conditions, suggesting that physical occlusion of divisome assembly is unlikely to be the sole mechanism by which Noc proteins mediate division inhibition. Our results suggest that an additional species-specific interaction between Noc and a divisome protein is also likely to be required for its activity. The search for this elusive interaction partner should therefore continue.

### Coordinating DNA-replication and cell division

To ensure faithful inheritance of genetic material, cell division must be coordinated with DNA replication and segregation. Noc is thought to contribute to this coordination by blocking cell division over the origin-proximal region of the chromosome where its binding sites are distributed [9]. This division inhibition activity is the only known function of *B*. *subtilis* Noc. However, as we have shown here, *S*. *aureus* Noc has the additional activity of restricting the initiation of DNA replication, thus further integrating the processes of division and DNA replication/segregation in this organism. To our knowledge, this activity for ^*Sa*^Noc is the first example of a ParB-like protein controlling replication initiation. ParB proteins typically bind centromeric *parS* sequences and work with ParA partner proteins to promote origin segregation [39]. In *B*. *subtilis*, the ParA protein Soj has also been shown to both inhibit and activate DNA replication initiation by DnaA in a manner modulated by the ParB protein Spo0J [29]. *S*. *aureus* lacks a ParA homolog, indicating that the effects on replication observed in mutants defective for ^*Sa*^Noc are not an indirect consequence of a misregulated ParA protein partner as is the case for *B*. *subtilis* mutants inactivated for Spo0J (ParB). Although we favor the idea that ^*Sa*^Noc directly modulates DnaA activity in *S*. *aureus* additional work is required to establish the mechanism of replication control.

Irrespective of the precise mechanism, our results raise the question of why *S*. *aureus* utilizes a Noc protein with the added activity of controlling the initiation of DNA replication whereas *B*. *subtilis* does not. Differences in cell morphology and the geometries of chromosome segregation and cell division in these organisms might explain the distinct Noc regulatory activities used by these organisms. In rod-shaped cells, chromosome segregation proceeds along the long cell axis. The spatial cues do not change from one cell cycle to the next. Therefore, even if replication were to outpace division, the pattern of chromosome segregation would remain the same. The situation in spherical cells like *S*. *aureus* is very different. Just as division alternates between three perpendicular planes, so too must the orientation of chromosome segregation. Thus, if replication were initiated prematurely relative to the progress of division, the appropriate spatial cues might not be in place to direct segregation in an orientation compatible with that of the next division plane. Furthermore, Noc-dependent inhibition of division due to the segregation of these prematurely replicated origins would be more difficult to overcome given the reduced spatial resolution in these small spherical cells. Consistent with this idea, we note that a significantly larger portion of the terminus region in *S*. *aureus* lacks Noc binding sites compared to *B*. *subtilis*. The dual function of ^*Sa*^Noc in nucleoid occlusion and replication control may therefore serve to maintain a tighter coupling between division and replication to keep the geometries of chromosome segregation in step with that of the division plane and/or to ensure that division is not impaired by prematurely segregated origins. The challenge for the future will be to determine how ^*Sa*^Noc restrains replication and how its apparent inhibitory effects are relieved in response to cell cycle signals to facilitate proper coordination between replication initiation and cell division.

## Materials and methods

### General methods

*S*. *aureus* strains were grown in tryptic soy broth (TSB) at 30°C or 37°C with aeration, unless otherwise indicated. The medium was supplemented with erythromycin (5 μg/ml for chromosomal insertions and 10 μg/ml for plasmids), chloramphenicol (5 μg/ml for chromosomal insertions, 10 μg/ml for plasmid), spectinomycin (100 μg/ml), kanamycin and neomycin (25 μg/ml each), 5-bromo-4-chloro-3-indolyl ß-D-galactopyranoside (X-Gal, 250 μg/ml), or anhydrotetracycline (aTc, 100 ng/ml unless otherwise indicated). *B*. *subtilis* strains were derived from the prototrophic strain PY79 [40]. Cells were grown in Luria Broth (LB) or Casein Hydrolysate (CH) medium [41] at 37°C with aeration, unless otherwise indicated. The medium was supplemented with tetracycline (10 μg/ml), spectinomycin (100 μg/ml), kanamycin 10 μg/ml), chloramphenicol (5 μg/ml), xylose (0.5% w/v) or isopropyl ß-d-thiogalactopyranoside (IPTG, 0.5 mM). Lists of oligonucleotide primers, strains, plasmids, and descriptions of their construction can be found in Supplemental Material.

### Transposon insertion library construction and sequencing

Transposon libraries of *S*. *aureus* strains TM18 (wt) and Δ*noc*::*spec* were constructed according to Wang *et al*. [19], except that the donor strain TM17 contained the transposon plasmid pTP77. Each library had >70,000 insertions. Transposon-sequencing (Tn-seq) was adapted from Zhang *et al*. [42]. Briefly, genomic DNA isolated from each library was sonicated (using Q800R2 QSONICA) to 200–400 bp fragments. Nicked ends were repaired (Quick Blunt kit, NEB), and dA-tails were added with Taq polymerase (NEB), followed by ligation of adapters using T4 ligase (NEB). Transposon-junctions were PCR-amplified using primers oTP182 and oTP183. PCR products were purified and incubated with the endonuclease NotI (NEB) to remove any contaminating DNA derived from the transposon plasmid. Finally, a second round of PCR was performed on 100 ng of each sample, using primers oTP184-187 and oTP188-191. A 200–400 bp product was gel-purified and sequenced on the Illumina HiSeq 2500 platform (Biopolymers Facility at Harvard Medical School).

The sequencing reads were mapped to each TA site on the *S*. *aureus* NCTC8325 genome (NCBI NC_007795.1). Genes in which reads were statistically underrepresented in Δ*noc* compared to wild-type were identified by the Mann Whitney *U* test. Visual inspection of transposon insertion profiles was performed with the Sanger Artemis Genome Browser and Annotation tool. Tn-seq datasets are accessible through NCBI's Gene Expression Omnibus under accession number GSE101335.

### Identification of permissive growth conditions

Noc depletion strains harboring Δ*rbd* or Δ*comEB* (strains aTP431 and aTP508) were streaked on plates containing 0.5 X LB No NaCl, LB No NaCl, LB 0.5% NaCl, or LB 1% NaCl, and incubated at 30˚, 37˚ or 42˚C. The colony size of each strain was compared with the Noc depletion strain aTP359 grown under the same conditions. The growth conditions in which colonies from aTP431 and aTP508 were comparable in size to aTP359 were considered permissive. LB 0.5% NaCl at 37°C did not allow growth for either aTP431 or aTP508 and was considered non-permissive.

### ChIP-Seq

ChIP was performed as described previously [43] with modification. Briefly, cells were grown in TSB medium to OD_600_ = 0.4 for *S*.*aureus*, and in CH medium to OD_600_ = 0.25 for *B*. *subtilis*, and incubated with 3% formaldehyde for 30 min at room temperature. Formaldehyde was quenched and the cells washed, and lysed. Lysates were sonicated using a Q800R2 sonicator (QSONICA) to generate 300–500 bp randomly sheared chromosomal DNA fragments. The lysates were then incubated with anti-^*Bs*^Noc or anti-6xHis antibodies (Genscript) overnight at 4°C. The sample was then processed as described previously and sequenced using the Illumina MiSeq platform. The sequencing reads were mapped to the *S*. *aureus* NCTC8325 genome (NCBI NC_007795.1) or *B*. *subtilis* PY79 genome (NCBI NC_022898.1) using CLC Genomics Workbench software (Qiagen). ChIP-seq datasets are accessible through Gene Expression Omnibus under accession number GSE93264.

### Whole genome sequencing

Whole genome sequencing was performed according to Baym *et al*. [44]. 1.5 ng genomic DNA from each strain was used in a 2.5 μl Nextera Tagmentation reaction (Illumina). After incubation at 37°C for 5 minutes, the reaction was immediately placed on ice. Tagmented DNA was then mixed with 11 μl KAPA Library Amplification Kit master mix (Kapa Biosystems), 4.4 μl of each indexing primer (5 μΜ) followed by PCR amplification. Amplified DNA was then purified from 15 μl of each reaction with 12 μl of Agencourt AMPure XP beads (Beckman Coulter) and resuspended in 30 μl buffer containing 10 mM Tris-Cl (pH 8.0), 1 mM EDTA (pH 8.0), and 0.05% Tween-20. The concentration of each sample was measured by Qubit (Thermo Fisher Scientific Inc.) and equivalent amounts of DNA were pooled, analyzed on TapeStation (Agilent Technologies) for quality control, and sequenced using the Miseq platform (Illumina). Sequencing reads were analyzed on CLC Genomics Workbench. Single nucleotide polymorphisms (SNP) and deletions were identified by comparing the sequence of the suppressors to the parental strains. Raw data are accessible through NCBI's Sequence Read Archive under accession number SRP111468.

### Fluorescence microscopy

Cells at OD_600_ ~ 0.4 were pelleted by centrifugation at 3,300 xg for 2 min, and immobilized on 2% (wt/vol) agarose pads containing M9 salts solution. Cell walls were stained with 0.5 μg/ml BODIPY FL vancomycin (Van-FL, ThermoFisher) and 0.5 μg/ml Vancomycin-HCl. Cell membranes were stained with 1 μg/ml Nile Red (ThermoFisher). DNA was stained with DAPI (Molecular Probes) at 2 μg/ml, or with Propidium Iodide (Molecular Probes) at 5 μM. To quantitatively characterize DNA content, cells were immediately fixed with ethanol followed by staining with Propidium iodide. Fluorescence microscopy was performed using a Nikon TE2000 inverted microscope with a Nikon CFI Plan Apo VC 100X objective lens. Images were cropped and adjusted using MetaMorph software (Molecular Devices). Final figures were prepared using Adobe Illustrator (Adobe Systems). Image analyses were performed using MATLAB. All cells were segmented using phase-contrast images. Well-separated cells that were not undergoing cytokinesis were manually selected and used for analysis. Cell volume and intensity of fluorescence signals were determined using the built-in functions in MATLAB.

### Electron microscopy

Cells were harvested at OD_600_ ~ 0.4, resuspended into LB medium, and fixed overnight at 4°C with 2.5% Glutaraldehyde, 1.25% Paraformaldehyde and 0.03% picric acid in 0.1 M sodium cacodylate buffer (pH 7.4). Fixed cells were washed in 0.1M cacodylate buffer and incubated with 1% Osmium Tetroxide (OsO4)/1.5% Potassium Ferrocyanide (KFeCN6) for 1 hour. Samples were then washed twice with ddH_2_O, washed once with Maleate buffer (MB), and incubated in 1% uranyl acetate in MB for 1 hour. Samples were washed twice with ddH_2_O, followed by dehydration in increasing concentration of alcohol (10 min each with 50%, 70%, 90%, twice with 100%). The samples were then incubated in propyleneoxide for 1 hour and infiltrated overnight in a 1:1 mixture of propyleneoxide and TAAB Epon (Marivac Canada Inc. St. Laurent, Canada). The samples were then embedded in TAAB Epon and polymerized at 60°C for 48 hrs. Ultrathin sections (~60 nm) were cut on a Reichert Ultracut-S microtome, mounted onto copper grids stained with lead citrate, and examined in a JEOL 1200EX Transmission electron microscope. Images were recorded with an AMT 2k CCD camera.

### Marker frequency analysis

*S*. *aureus* cells were grown in LB 0.5% NaCl medium at 37°C, except Δ*noc* Δ*rbd*, which was first grown under permissive conditions (0.5X LB no NaCl, 30°C) to OD_600_ ~0.4, then back diluted into nonpermissive conditions (LB 0.5% NaCl medium, 37°C) at an OD_600_ ~0.04. Sodium azide was added to cells at OD_600_ ~0.4 to prevent further growth and chromosomal DNA was then isolated using DNeasy Blood and Tissue Kit (Qiagen). To obtain cells with an *ori*:*ter* ratio of 1, exponentially growing cells (OD_600_ = 0.15) were treated with 50 μg/ml rifampicin for 1 hour at 37°C before harvesting. Quantitative PCR was performed using the SYBR FAST qPCR Kits (Kapa Biosystems) on a StepOne Plus Real-Time PCR System (Applied Biosystems). Primer sets oTP478/oTP479 and oTP480/oTP481 were used to amplify the *S*. *aureus oriC* and *ter* regions, respectively. The 2-ΔΔCt method was used to quantify the relative *ori*:*ter* ratio, where chromosomal DNA sample from rifampicin-treated wild-type cells was used for normalization. Marker-frequency analysis of *B*. *subtilis* strains was the same as *S*. *aureus*, except that cells were grown in CH medium at 30°C until OD_600_ ~ 0.25. Primer sets oTP482/oTP483 and oTP484/oTP485 were used to amplify the *B*. *subtilis oriC* and *ter* regions, respectively.

Cell growth and chromosomal DNA preparation were the same for genome-wide marker frequency analysis. Whole genome sequencing was performed as described above. The sequencing reads from all strains were normalized to 51 million and the data were plotted in 30 kb bins. The smoothed conditional means were determined by LOESS method using ggplot2 in R [45]. The fraction of points used to fit each local regression (spanS) was 0.08. WGS data for marker frequency analysis are accessible through Gene Expression Omnibus under accession number GSE100776.

### Immunoblot analysis

Whole cell lysates from exponentially growing cultures (OD_600_ = 0.4) were prepared as previously described [46]. Samples were heated at 100°C for 5 min prior to loading. Equivalent amount of cells based on OD_600_ at the time of harvest were separated by SDS-PAGE on Tris-Glycine gels, and transferred to PVDF membrane (EMD Millipore). Membranes were blocked in 5% nonfat milk with 0.5% Tween-20 for 1 h. Blocked membranes were probed with anti-6xHis (1:4000) (Genscript), anti-Noc (*B*. *subtilis*) (1:10,000), anti-DnaA (*B*. *subtilis*) (1:10,000) [47], or anti-σ^A^ (*B*. *subtilis*) (1:10,000) [48] antibodies. Primary antibodies were detected with horseradish-peroxidase conjugated anti-mouse (for anti-His) or anti-rabbit (for anti-Noc, anti-DnaA and anti-σ^A^) antibodies (BioRad) and detected with Western Lightning ECL reagent as described by the manufacturer. Images were obtained by using the Molecular Imager Gel Doc XR system (Bio-Rad) and analyzed by ImageJ [49]. *S*. *aureus* immunoblots were performed in a strain lacking the *spa* gene encoding Surface Protein A.

## Supporting information

S1 Text Supplemental materials and methods. (DOCX)

S1 Table Candidate *sln* genes identified by Tn-seq. (DOCX)

S2 Table Quantification of abnormal septa and lysis in cells lacking Noc and Rbd. (DOCX)

S3 Table Δ*noc* Δ*rbd* and Δ*noc* Δ*comEB* suppressors. (DOCX)

S4 Table Strains used in this study. (DOCX)

S5 Table Plasmids used in this study. (DOCX)

S6 Table Oligonucleotides primers used in this study. (DOCX)

S7 Table Peaks enriched by ChIP-seq in *S*. *aureus* using anti-^*Bs*^Noc antibody. (DOCX)

S8 Table Tn-seq results. (XLSX)

S1 Fig Abnormal septa and lysis in *S*. *aureus* cells lacking Noc and Rbd. Electron micrographs of HG003 (wt) and indicated mutants. Overnight cultures were grown under permissive condition (half-strength LB without NaCl) at 30°C to an OD_600_ of 0.5. Cells were then washed, and diluted to an OD_600_ of 0.02 in LB with 0.5% NaCl, and grown at 37°C for ~4 mass doublings. Cells were then harvested, processed, and visualized by electron microscopy as described in the Methods. Examples of abnormal septa (blue arrows) and lysed cells (red arrows) are highlighted. Scale bar indicates 1 μm.
(TIF)

S2 Fig Permissive and non-permissive growth conditions for Δ*noc* Δ*rbd* and Δ*noc* Δ*comEB*. Spot dilutions of the indicated HG003 strains grown under different conditions. The Noc depletion strains (*P*_*tet*_-*noc*) were grown overnight in liquid medium under the conditions indicated by the red boxes. Wild-type (wt) and Δ*noc* were grown in LB with 0.5% NaCl. Saturated cultures were washed and diluted to an OD_600_ of 0.2 and 10-fold serially diluted. 5 μl of each dilution were spotted onto the indicated plates, incubated overnight, and photographed.
(TIF)

S3 Fig Characterization of Noc in replication control. (**A**). Analysis of DNA content relative to cell volume in wt and derivatives. The indicated strains grown under the same conditions as described in Fig 4B were fixed with ethanol and later stained with the fluorescent DNA dye propidium iodide (PI) and examined by phase contrast and fluorescence microscopy. The Fluorescence intensity (mean ± standard deviation) and cell volume (mean ± standard deviation) were quantified from fluorescence and phase contrast images (n>100), and plotted on the graph. The ratio of fluorescence intensity to cell volume (FI/Vol) of each strain is shown. *p* ≤ 0.0001 (****) *p* > 0.05 (ns). (**B**). The plots show the ratios of the indicated genomic profiles. Overnight cultures of *S*. *aureus* strain RN4220 (wt) and Δ*noc* cells expressing *noc* under the control of the *P*_*tet*_ promoter were diluted to OD_600_ = 0.01 and grown in TSB medium without or with inducer (aTc) at 37°C. Genomic DNA was isolated after 5 mass doublings and analyzed by whole-genome sequencing. The total sequencing reads from each strain were normalized to 51 million and the data were plotted relative to the sequencing reads of the first biological replicate of wild-type. Circles show 30 kb bins. Blue lines and grey area represent the smoothed conditional mean and 95% confidence band for the regression curve, respectively (spanS = 0.08). The data from one of two biological replicates are shown. The left plot is identical to the one presented in Fig 4C and is included to facilitate a direct comparison.
(TIF)

S4 Fig Genome-wide marker frequency analysis after inhibition of cell division. (**A**). The plots show the ratios of genomic profiles from Δ*noc* and wild-type (wt) before and after treatment with the FtsZ inhibitor PC190723. Overnight cultures of *S*. *aureus* strain RN4220 (wt) and Δ*noc* were diluted to OD_600_ ~ 0.01 and grown in TSB medium at 37°C. The inhibitor PC190723 (2μg/ml) was added to each culture at OD_600_ = 0.13. Cultures were harvested before (0h) and 0.5h or 1.5h following the addition of the drug. The total sequencing reads from each strain were normalized to 51 million and the data were plotted as a ratio of Δ*noc* to wild-type at each time point. Circles show 30 kb bins. Blue lines and grey area represent the smoothed conditional mean and 95% confidence band for the regression curve, respectively (spanS = 0.08). The data from one of two biological replicates are shown. (**B**). Increase in DNA content and volume after treatment with PC190723. Cells treated the same way as in (A) were fixed with ethanol and later stained with fluorescent DNA dye propidium iodide (PI) and examined by phase contrast and fluorescence microscopy. The Fluorescence intensity (mean ± standard deviation) and cell volume (mean ± standard deviation) were quantified from fluorescent and phase contrast images (n>100), and plotted on the graph. Inset shows the growth curves for the cells used for the cytological analysis. PC190723 was added at time 0h.
(TIF)

S5 Fig Representative images of cells used for analysis in S4B Fig. Cultures were harvested before (0h) and at 0.5h or 1.5h following the addition of PC190723 (2μg/ml), fixed with ethanol and later stained with fluorescent DNA dye propidium iodide (PI). Cells were then visualized by phase contrast (Phase) and fluorescence (PI) microscopy. Scale bar indicates 1 μm.
(TIF)

S6 Fig ^*Sa*^Noc-YFP and ^*Bs*^Noc-YFP localize to the peripheral membranes in *B*. *subtilis*. Larger fields of *B*. *subtilis* Δ*noc* mutants with ^*Bs*^*noc-yfp* or ^*Sa*^*noc-yfp* expressed under the control of a xylose-inducible promoter. Cells were induced at OD_600_ = 0.01 with 0.5% xylose and analyzed by fluorescence microscopy at OD_600_ = 0.25. Scale bar indicates 1 μm.
(TIF)

S7 Fig Noc enrichment profiles as determined by ChIP-Seq. The indicated *S*. *aureus* strains were grown to OD_600_ = 0.4, and processed as described in the Methods. Δ*noc* (HG003) strains harboring *P*_*tet*_ fusions to *his*-tagged *B*. *subtilis noc* (^*Bs*^*noc*_*his*_) or *S*. *aureus noc* (^*Sa*^*noc*_*his*_) were induced at OD_600_ = 0.05 and treated with formaldehyde at an OD_600_ = 0.4. Wild-type (wt) (HG003) and Δ*noc* were grown without inducer but were cross-linked and processed identically to the *his*-tagged strains. ChIP-seq was performed with anti-6XHis antibodies. The profiles were generated by normalizing the reads from the ChIP-Seq samples to those from input genomic DNA. The data are plotted in 1 kb bins. The higher background and higher peaks in the ^*Bs*^Noc_*his*_ ChIP-seq profile are due to fewer and sparser sequencing reads in this sample and the normalization process used. The origin is at position 1 with positions on the left and right chromosome arms indicated as negative or positive numbers, respectively.
(TIF)

S8 Fig ^*Bs*^Noc_*his*_ and ^*Sa*^Noc_*his*_ bind the same sites in *B*. *subtilis*. ChIP-seq using the anti-6xHis antibodies was performed on *B*. *subtilis* Δ*noc* cells expressing ^*Bs*^*noc*_*his*_ or ^*Sa*^*noc*_*his*_ under the control of the *P*_*spank*_ promoter. Wild-type and Δ*noc* cells lacking a *his*-tagged *noc* fusion were used as negative controls. Data presented were first normalized to the total number of reads then relative to the input for each strain and finally relative to that of the relevant control sample (wild-type cells for ^*Bs*^*noc*_*his*_ and Δ*noc* cells for ^*Sa*^*noc*_*his*_). The data are plotted in 1 kb bins. Genome positions are labeled as in S7 Fig.
(TIF)

S9 Fig Increased cell size in the *S*. *aureus* Δ*noc* mutant overexpressing DnaA. Representative fields of wild-type (HG003) or Δ*noc* strains harboring an empty vector or the same plasmid with *dnaA* fused to a *P*_*tet*_ promoter. Overnight cultures were diluted to an OD_600_ = 0.01 then grown to an OD_600_ of 0.05 at which time the indicated amount of inducer was added. Cells were harvested at OD_600_ = 0.4, washed and resuspended in phosphate buffered saline (1XPBS) with BODIPY FL-Vancomycin (Van-FL), and examined by phase contrast (Phase) and fluorescence (Van-FL) microscopy. Scale bar indicates 1 μm. Average cell volumes (μm^3^) ± standard deviations (n>200) are indicated below the micrographs.
(TIF)

S10 Fig *dnaA* mutations partially suppress the large cell phenotype of the Δ*noc* mutant. Representative fluorescent images of HG003 (wt) and indicated derivatives. Overnight cultures were diluted to OD_600_ = 0.01 in LB 0.5% NaCl at 37°C. When cultures reached OD_600_ = 0.4, cells were washed and resuspended in Phosphate buffered saline (1XPBS) with the BODIPY FL-Vancomycin (Van-FL) and DAPI and examined by phase contrast (Phase) and fluorescence microscopy. Scale bar indicates 1 μm. Average cell volumes (μm^3^) ± standard deviations (n>120) are shown below the micrographs. DAPI-stained cells contain a representative example of a strong-staining Δ*noc* cell that suggested increased cell envelope permeability in the mutant.
(TIF)

S11 Fig Genomic profiling of a *dnaC* suppressor of Δ*noc*Δ*rbd*. Genome-wide DNA content of the indicated strains. Overnight cultures of *S*. *aureus* strains HG003 (wt) and Δ*noc*Δ*rbd dnaC*_*A352V*_ were diluted to OD_600_ = 0.01 and grown in LB 0.5% NaCl medium at 37°C. The Δ*noc*Δ*rbd* strain was first grown under permissive conditions (0.5X LB no NaCl, 30°C) to OD_600_ ~0.4, then back diluted into nonpermissive conditions (LB 0.5% NaCl medium, 37°C) at an OD_600_ ~0.04. All cultures were harvested at OD_600_ ~0.4. Genomic DNA was isolated and analyzed by whole-genome sequencing as described in Fig 4B. Data were normalized to 51 million reads and plotted in 30 kb bins (circles). Blue lines and grey area represent the smoothed conditional mean and 95% confidence band for the regression curve, respectively (spanS = 0.08). The data from one of two biological replicates are shown. *ori*:*ter* ratios indicated below the plots were determined using the 30kb bins spanning the origin and terminus.
(TIF)
