# Supplementary material for: The nucleoid occlusion factor Noc controls DNA replication initiation in Staphylococcus aureus
Source: PLoS Genet. 2017 Jul 19;13(7):e1006908. doi: 10.1371/journal.pgen.1006908 (PMC5540599; doi:10.1371/journal.pgen.1006908)
Supplement: S1 Text — (DOCX) [file pgen.1006908.s001.docx]

**S1 Text. Supplemental Materials and Methods**

**Plasmid construction**

**pTP016 [allelic replacement plasmid]** was constructed by inserting annealed oligos oTP048 and oTP049 into pTP009 between BamHI and NcoI. pTP009 was constructed by isothermal assembly from three PCR products. Product 1 was derived from pMAD [1] (amplified with oTP023 and oTP024) and contained the pBR322 origin, *bla*, and *erm*. Product 2 was derived from pBursa [2] (amplified with oTP025 and oTP026) and contained the temperature-sensitive origin pE194ts. Product 3 was derived from pMAD (amplified with oTP027 and oTP028) and contained *lacZ*.

**pTP078 [∆*noc*::*spec* insertion-deletion plasmid]** was constructed by isothermal assembly using three PCR products and the allelic replacement vector pTP016 cut with EcoRI and EagI. Product 1 was derived from pWX466 (amplified with oTP046 and oTP047) and contained the *spec* gene. Product 2 is the 1 kb region of DNA upstream of the *noc* gene (amplified from HG003 gDNA using oTP120 and oTP200). Product 3 is the 1 kb region of DNA downstream of the *noc* gene (amplified from HG003 gDNA using oTP201 and oTP123). pWX466 is a plasmid harboring the *spec* gene flanked by *loxP* sites (XW and DZR, unpublished).

**pTP083 [∆*parB*::*kan* insertion-deletion plasmid]** was constructed by isothermal assembly using three PCR products and the allelic replacement vector pTP016 cut with EcoRI and EagI. Product 1 was derived from pWX470 (amplified with oTP046 and oTP047) and contained the *kan* gene. Product 2 is the 1 kb region of DNA upstream of the *parB* gene (amplified from HG003 gDNA using oTP226 and oTP227). Product 3 is the 1 kb region of DNA downstream of the *parB* gene (amplified from HG003 gDNA using oTP228 and oTP229). pWX470 is a plasmid harboring the *kan* gene flanked by *loxP* sites (XW and DZR, unpublished).

**pTP088 [∆*rbd*::*kan* insertion-deletion plasmid]** was constructed by isothermal assembly using three PCR products and the allelic replacement vector pTP016 cut with EcoRI and EagI. Product 1 was derived from pWX470 (amplified with oTP046 and oTP047) and contained the *kan* gene. Product 2 is the 1 kb region of DNA upstream of the *rbd* gene (amplified from HG003 gDNA using oTP244 and oTP245). Product 3 is the 1 kb region of DNA downstream of the *rbd* gene (amplified from HG003 gDNA using oTP246 and oTP247).

**pTP095 [∆*comEB*:*kan* insertion-deletion plasmid]** was constructed by isothermal assembly using three PCR products and the allelic replacement vector pTP016 cut with EcoRI and EagI. Product 1 was derived from pWX470 (amplified with oTP046 and oTP047) and contained the *kan* gene. Product 2 is the 1 kb region of DNA upstream of the *comEB* gene (amplified from HG003 gDNA using oTP265 and oTP266). ). Product 3 is the 1 kb region of DNA downstream of the *comEB* gene (amplified from HG003 gDNA using oTP267 and oTP268).

**pTP077 [transposon containing plasmid with NotI sites]** was constructed by isothermal assembly from two PCR products. Product 1 was amplified from pTM402 [3] using oTP196 and 197. Both oligonucleotide primers containined NotI sites. Product 2 was amplified from pTM402 using oTP198 and oTP199.

**pTP044** **[L54a integrase expression plasmid]** was constructed by isothermal assembly from two DNA fragments. Fragment 1 was a PCR product derived from pFA545 [2] (amplified using oTP096 and oTP097) that contained the *repC* and *tetR* region. Fragment 2 was derived from pYL112d19 [4] (digested with StuI and ApaI) and contained the *bla* and L54a *int* gene.

**pTP069** **[*P_tet_*-*^Sa^noc S. aureus* integration plasmid]** was generated in a 2-way ligation with a PCR product containing the *S. aureus* *noc* gene with its native RBS (amplified from HG003 gDNA using oTP126 and oTP127) and plasmid pTP063 digested with HpaI and EcoRI. pTP063 (*P_tet_* integration vector) was generated by replacing the *P_spank_* promoter and *lacI* gene from pTP041 with the *P_xyl/tet_* promoter, *tetR*, and *lacZ* region from pRAB14-*lacZ* (both plasmids were digested with EcoRI and HindIII). pTP041 (*P_spank_* integration vector) was generated by isothermal assembly from four PCR products. Product 1 was derived from pDR110 (amplified using oTP078 and 090) contained the *P_spank_* promoter and *lacI*. Product 2 was derived from pWX465 (amplified with oTP088 oTP089) contained the *cat* gene. Product 3 derived from pLL29 (amplified using oTP091 and oTP083) contained *attP*(L54a). Product 4 derived from pML6 (amplified with oTP084 and oTP085) contained the pACYC origin and *bla*. pRAB14-*lacZ* is a *S. aureus* expression vector containing *tetR*-*P_xyl/tet_* [5]. pLL29 is an integration vector containing *attP*(L54a) [6]. pDR110 contains the *P_spank_* promoter (DZR unpublished). pWX465 is a plasmid harboring the *cat* gene flanked by *loxP* sites (XW and DZR unpublished). pML6 is a vector containing the pACYC origin and *bla gene* (PML and DZR unpublished).

**pTP167** **[*P_tet_*-(optimized RBS) *^Bs^noc S. aureus* integration plasmid]** was constructed by site-directed mutagenesis using oligos oTP441 and oTP442 (containing a consensus RBS) and plasmid pTP144. pTP144 [*P_tet_*-*^Bs^noc*] was constructed by isothermal assembly of a PCR product containing the *B. subtilis noc* gene (amplified from PY79 gDNA using oTP414 and oTP415) and pTP063 digested by HpaI and EcoRI.

**pTP170** **[*P_tet_*-*^Sa^noc_his_ S. aureus* integration plasmid]** was constructed by isothermal assembly of a single PCR product from plasmid pTP069 using oTP445 and oTP310 (containing a His6 tag). The PCR product was treated with DpnI to degrade the template DNA prior to isothermal assembly.

**pTP171** **[*P_tet_*-(optimized RBS) *^Bs^noc_his_ S. aureus* integration plasmid]** was constructed by isothermal assembly of a single PCR product from plasmid pTP167 using oTP446 and oTP310 (containing a His6 tag). The PCR product was treated with DpnI to degrade the template DNA prior to isothermal assembly.

**pTP137** **[*ycgO*::*P_spank_*- *^Bs^noc spec*]** was constructed in a 2-way ligation with a PCR product containing the *B. subtilis* *noc* gene with its native RBS (amplified from PY79 gDNA using oTP389 and oTP402) and pER107 digested with NheI and XmaI. pER107 is an ectopic integration vector containing the *P_spank_* promoter for insertions in the nonessential *ycgO* gene (ER and DZR, unpublished).

**pTP169** **[*ycgO*::*P_spank_*-(optimized RBS) *^Sa^noc* *spec*]** was constructed by site-directed mutagenesis using oTP439 and oTP440 (containing a consensus RBS) and pTP136. pTP136 was constructed in a 2-way with a PCR product containing the *S. aureus noc* gene with its native RBS (amplified from HG003 gDNA using oTP387 and oTP401) and pER107 digested with NheI and XmaI.

**pTP173** **[*ycgO*::*P_spank_*- *^Bs^noc_his_ spec*]** was constructed by isothermal assembly of a single PCR product from plasmid pTP137 using oTP446 and oTP447. The PCR product was treated with DpnI to degrade the template DNA prior to isothermal assembly.

**pTP174** **[*ycgO*::*P_spank_*-(optimized RBS) *^Sa^noc_his_ spec*]** was constructed by isothermal assembly of a single PCR product from plasmid pTP169 using oTP445 and oTP447. The PCR product was treated with DpnI to degrade the template DNA prior to isothermal assembly.

**pTP200 [*amyE*::*P_xylA_*-(optimized RBS) *^Sa^noc*-*yfp* spec]** was constructed by isothermal assembly using the ectopic integration vector pDR150 digested with BamHI and EcoRI and a PCR product amplified from an isothermal assembly reaction using oTP 501 and oTP511. The isothermal assembly reaction contained three PCR products. Product 1 derived from pDR150 (amplified with oTP501 and oTP510) contained the *P_xyl_* and *xylR*. Product 2 containing *yfp* was amplified from gDNA extracted from strain bKM1585 using oTP502 and oTP511. Product 3 containing *^Sa^noc* with an optimized RBS was amplified from pTP174 with oTP505 and oTP508. pDR150 is an ectopic integration vector containing the *P_xyl_* promoter for insertions in the nonessential *amyE* gene (DZR, unpublished).

***S. aureus* strain construction**

**Construction of the knock-out mutants**

*S. aureus* knock-out mutants were generated by allelic replacement. pMAD-based plasmids (pTP078, 083, 088, 095) were transformed into strain RN4220 (for pTP083, 088, 095) or TM18 (for pTP078), followed by selection on TSB plates containing erythromycin (10 µg/ml) at 30˚C. After incubation for 2 days, transformants were streaked on TSB plates containing erythromycin (10 µg/ml) and X-gal (250 µg/ml). One blue colony was inoculated in liquid TSB medium supplemented with erythromycin (10 µg/ml), and incubated with shaking for 3 h at 30˚C followed by 6 h at 37˚C. 100ul of a 20-fold dilution was then plated onto TSB plate containing erythromycin (10 µg/ml) and X-gal (250 µg/ml) and incubated overnight at 37˚C to generate single-crossover integrants. A single blue colony was then inoculated into TSB medium without antibiotics and grown at 30˚C. After the culture became turbid, cells were diluted 30-fold into the same medium and grown again at 30˚C. After the culture became turbid, cells were 30-fold diluted and grown at 30˚C for 3 h, followed by 37˚C for 6 h. Serial dilutions of this culture were then plated on TSB supplemented with X-Gal and the antibiotic corresponding to the antibiotic resistance cassette used to replace the target gene (kanamycin/neomycin for ∆*comEB,* ∆*rbd,* ∆*parB*, and spectinomycin for ∆*noc*). White colonies were then tested for erythromycin sensitivity. The insertion-deletions were then confirmed by colony PCR using primers amplifying a region 1 kb upstream and downstream of the target gene. The insertion-deletions were transduced into HG003 using phage 80alpha.

**Construction of complementation strains**

HG003 (∆*noc*::*spec*, *geh*::pTP069) was constructed by phage transduction using *S. aureus* phage 80alpha infected RN4220 (*geh*::pTP069) as donor, and HG003 (∆*noc*::*spec*) as recipient. RN4220 (*geh*::pTP069) was constructed by electroporating plasmid pTP069 into strain RN4220 (pTP044), and selection on TSB plate containing chloramphenicol (5 µg/ml). In the presence of L54a integrase, expressed from pTP044, pTP069 integrates into the *attB*(L54a) site within the *geh* gene of *S. aureus*. RN4220(∆*noc*::*spec*, *geh*::pTP069) was constructed in the same way, except that RN4220(∆*noc*::*spec*) was used as recipient.

HG003 (∆*noc*::*spec*, *geh*::pTP170), and HG003 (∆*noc*::*spec*, *geh*::pTP171) were constructed in the same way as HG003 (∆*noc::spec*, *geh*::pTP69).

HG003 (∆*noc*::*spec*, ∆*rbd*::*kan*, *geh*::pTP069) and HG003 (∆*noc*::*spec*, ∆*comEB*::*kan*, *geh*::pTP69) were constructed by phage transduction using phage 80alpha infected RN4220 (∆*rbd*::*kan*) or RN4220 (∆*comEB*::*kan*) as donor, and HG003 (∆*noc*::*spec*, *geh*::pTP69) as recipient.

HG003 (∆*noc*::*spec*, ∆*rbd*::*kan*) and HG003 (∆*noc*::*spec*, ∆*comEB*::*kan*) were constructed by phage transduction using phage 80alpha infected RN4220 (∆*rbd*::*kan*) or RN4220 (∆*comEB*::*kan*) as donor, and HG003 (∆*noc*::*spec*) as recipient. Transductants of HG003 (∆*noc*::*spec*, ∆*rbd*::*kan*) were selected on agar plates with 0.5X LB lacking NaCl supplemented with kanamycin/neomycin (25 µg/ml each), and incubated at 30°C for 2 days. Transductants of HG003 (∆*noc*::*spec*, ∆*comEB*::*kan*) were selected on agar plates with LB lacking NaCl supplemented with kanamycin/neomycin, and incubated at 37°C overnight.

**Reconstruction of suppressor mutants**

HG003 (∆*noc*::*spec* *dnaA*^sup1^), HG003 (∆*noc*::*spec* *dnaA*^sup2^), and RN4220 (∆*noc*::*spec* *dnaA*^sup1^) were constructed by phage transduction using phage 80alpha. The originally suppressor strains aTP512 HG003 (∆*noc*::*spec* ∆*rbd*::*kan* *dnaA*^sup1^) and aTP522 HG003 (∆*noc*::*spec* ∆*rbd*::*kan* *dnaA*^sup2^) were used as donors and HG003 or RN4220 as recipient. Transductants were selected on TSB plates supplemented with spectinomycin, and screened for kanamycin sensitivity. To screen for transductants that contained the linked *dnaA* suppressor mutation, colony PCR was performed on the transductants using oTP342 and oTP343 and the products were purified and sequenced using oTP342.

Reconstructed suppressor strains aTP774 HG003 (∆*noc*::*spec* ∆*rbd*::*kan* *dnaA*^sup1^) and aTP776 HG003 (∆*noc*::*spec* ∆*rbd*::*kan* *dnaA*^sup2^) were constructed in the same way as HG003 (∆*noc*::*spec* *dnaA*^sup1^), except that HG003 (∆*rbd*::*kan*) was used as recipient, and that transductants were selected on agar plates with 0.5X LB lacking NaCl supplemented with spectinomycin at 30°C.

**Construction of strains for immunoblot and ChIP-seq**

Strains used for immunoblot analsyis and ChIP-seq contained a transposon insertion in the *spa* gene encoding Surface Protein A. The donor strain was NE286, a USA300 strain harboring a mariner transposon insertion in *spa* [7]. The transducing phage used was phage 80alpha. Transductants were selected on TSB plates supplemented with 5 ug/ml erythromycin.

**Construction of strains for Tn-Seq validation**

Transposon insertions from the Nebraska Transposon Mutant Library [7] were first transduced into HG003 and then into the Noc depletion strain (aTP359) using phage 80alpha selecting for erythromycin resistance.

**Construction of *Bacillus subtilis* strains**

bTP039 [∆*noc*::*tet*, *ycgO*::P*_spank_*-*^Bs^noc_his_ spec*] and bTP041 [∆*noc*::*tet*, *ycgO*::P*_spank_*-*^Sa^noc_his_ spec*] were constructed by transforming gDNA from bRB73 (∆noc::*tet*) into strains bTP035 (*ycgO*::P*_spank_*-*^Bs^noc_his_ spec*) and bTP037 ([∆*noc*::*tet*, *ycgO*::P*_spank_*-*^Sa^noc_his_ spec*). bTP035 and bTP037 were constructed by transforming SacII-digested plasmid pTP173 and pTP174 into (*ycgO*::*cat*) selecting for Spec(R) and screening for Cm(S).

bTP043 [∆*noc*::*tet* ∆*minD*::*kan* *ycgO*::*P_spank_*-*^Bs^noc_his_* *spec*] and bTP045 [∆*noc*::*tet* ∆*minD*::*kan* *ycgO*::*P_spank_*-*^Sa^noc_his_* *spec*] were constructed by transforming gDNA from bML712 (∆*noc*::*tet* ∆*minD*::*kan*) into strains bTP039 and bTP041 selecting for Kan(R) and screening for Tet(R) and Spec(R).

bTP061 [∆*noc*::*tet* *amyE*::*P_xyl_*-*^Sa^noc-yfp spec*] was constructed by transforming the SacII-digested plasmid pTP200 into PY79 ∆*noc*::*tet* selecting for Spec(R) and screening for the inability to degrade starch.

**Supplemental References**

1. Arnaud M, Chastanet A, Debarbouille M (2004) New vector for efficient allelic replacement in naturally nontransformable, low-GC-content, gram-positive bacteria. Appl Environ Microbiol 70: 6887-6891.

2. Bae T, Banger AK, Wallace A, Glass EM, Aslund F, et al. (2004) Staphylococcus aureus virulence genes identified by bursa aurealis mutagenesis and nematode killing. Proc Natl Acad Sci U S A 101: 12312-12317.

3. Wang H, Claveau D, Vaillancourt JP, Roemer T, Meredith TC (2011) High-frequency transposition for determining antibacterial mode of action. Nat Chem Biol 7: 720-729.

4. Lee CY, Buranen SL, Ye ZH (1991) Construction of single-copy integration vectors for Staphylococcus aureus. Gene 103: 101-105.

5. Helle L, Kull M, Mayer S, Marincola G, Zelder ME, et al. (2011) Vectors for improved Tet repressor-dependent gradual gene induction or silencing in Staphylococcus aureus. Microbiology 157: 3314-3323.

6. Luong TT, Lee CY (2007) Improved single-copy integration vectors for Staphylococcus aureus. J Microbiol Methods 70: 186-190.

7. Fey PD, Endres JL, Yajjala VK, Widhelm TJ, Boissy RJ, et al. (2013) A genetic resource for rapid and comprehensive phenotype screening of nonessential Staphylococcus aureus genes. MBio 4: e00537-00512.

8. Nair D, Memmi G, Hernandez D, Bard J, Beaume M, et al. (2011) Whole-genome sequencing of Staphylococcus aureus strain RN4220, a key laboratory strain used in virulence research, identifies mutations that affect not only virulence factors but also the fitness of the strain. J Bacteriol 193: 2332-2335.

9. Herbert S, Ziebandt AK, Ohlsen K, Schafer T, Hecker M, et al. (2010) Repair of global regulators in Staphylococcus aureus 8325 and comparative analysis with other clinical isolates. Infect Immun 78: 2877-2889.

10. Youngman PJ, Perkins JB, Losick R (1983) Genetic transposition and insertional mutagenesis in Bacillus subtilis with Streptococcus faecalis transposon Tn917. Proc Natl Acad Sci U S A 80: 2305-2309.

11. Wu LJ, Ishikawa S, Kawai Y, Oshima T, Ogasawara N, et al. (2009) Noc protein binds to specific DNA sequences to coordinate cell division with chromosome segregation. EMBO J 28: 1940-1952.

12. Kurokawa K, Mizumura H, Takaki T, Ishii Y, Ichihashi N, et al. (2009) Rapid exchange of bound ADP on the Staphylococcus aureus replication initiation protein DnaA. J Biol Chem 284: 34201-34210.

13. Liew AT, Theis T, Jensen SO, Garcia-Lara J, Foster SJ, et al. (2011) A simple plasmid-based system that allows rapid generation of tightly controlled gene expression in Staphylococcus aureus. Microbiology 157: 666-676.
