# Supplementary material for: The nucleoid occlusion factor Noc controls DNA replication initiation in Staphylococcus aureus
Source: PLoS Genet. 2017 Jul 19;13(7):e1006908. doi: 10.1371/journal.pgen.1006908 (PMC5540599; doi:10.1371/journal.pgen.1006908)
Supplement: S1 Table — (DOCX) [file pgen.1006908.s002.docx]

**S1 Table** Candidate *sln* genes identified by Tn-seq

| Gene name | Annotation | p-value | Fold-change | Validation |
| --- | --- | --- | --- | --- |
| *comEB* | deoxycytidylate deaminase | 0.06 | 17.5 | +++ |
| *mprF* | lysyl-phosphotidylglycerol synthase | 0.007 | 9.3 | - |
| SAOUHSC_01496 | cytidylate kinase | 0.001 | 7.5 | N/A |
| SAOUHSC_01270 | putative uncharacterized protein | 0.35 | 7.5 | - |
| *yabA* | regulator of DNA replication initiation | 0.49 | 7.4 | - |
| *ecsB* | putative ABC transporter | 0.028 | 6.8 | + |
| SAOUHSC_01154 | SepF cell division protein | 0.021 | 6.8 | N/A |
| SAOUHSC_02337 | MurA peptidoglycan precursor biosynthesis | 0.046 | 6.7 | - |
| *rbd* | rhomboid protease, putative | 0.07 | 6.3 | +++ |
| SAOUHSC_01497 | L-asparaginase, putative | 0.10 | 6.1 | - |
| *tagA* | techoic acid biosynthesis | 0.010 | 5.6 | N/A |
| SAOUHSC_01050 | putative uncharacterized protein | 0.013 | 3.8 | + |
| *fmtA* | Affects methicillin resistance level | 0.038 | 3.6 | N/A |
