# Supplementary material for: The nucleoid occlusion factor Noc controls DNA replication initiation in Staphylococcus aureus
Source: PLoS Genet. 2017 Jul 19;13(7):e1006908. doi: 10.1371/journal.pgen.1006908 (PMC5540599; doi:10.1371/journal.pgen.1006908)
Supplement: S2 Table — (DOCX) [file pgen.1006908.s003.docx]

**S2 Table** Quantification of abnormal septa and lysis in cells lacking Noc and Rbd

|  | total cells counted | percent with abnormal septa | percent lysed cells |
| --- | --- | --- | --- |
| WT | 724 | 1.1% | 0 |
| Δ*noc* | 310 | 4.2% | 4.5% |
| Δ*rbd* | 507 | 5.9% | 0.6% |
| Δ*noc* Δ*rbd* | 353 | 27.2% | 23.8% |
