# Supplementary material for: The nucleoid occlusion factor Noc controls DNA replication initiation in Staphylococcus aureus
Source: PLoS Genet. 2017 Jul 19;13(7):e1006908. doi: 10.1371/journal.pgen.1006908 (PMC5540599; doi:10.1371/journal.pgen.1006908)
Supplement: S3 Table — (DOCX) [file pgen.1006908.s004.docx]

**S3** **Table** ∆*noc* ∆*rbd* and ∆*noc* ∆*comEB* suppressors

| **strain** | **mapped reads** | **loci** | **aa change** | **confirmed** |
| --- | --- | --- | --- | --- |
| Δ*noc*Δ*rbd* | 664,476 |  |  |  |
| #2 | 935,976 | Deletion in 5’ UTR of *dnaA* |  | Yes |
| #3 | 1,877,640 | SAOUHSC_01907 Aldo-keto reductase (AKRs) superfamily  Point mutation upstream of SAOUHSC_R0005 | N10S  N/A |  |
| #4 | 257,669 | No mutation detected |  |  |
| #5 | 1,260,537 | SAOUHSC_00018: DnaC helicase | A352V | Yes |
| #6 | 787,308 | upstream of SAOUHSC_01866 | N/A |  |
| #7 | 2,723,259 | *dnaA* | V141L | Yes |
| #8 | 1,846,980 | *pheS*: phenylalanyl-tRNA synthetase subunit alpha | M48K | Yes |
| #9 | 3,067,565 | rRNA: Sa5SA, Gene: SAOUHSC_R00011 | N/A |  |
| #10 | 2,172,921 | *aroA*: 3-phosphoshikimate 1-carboxyvinyltransferase | T291frameshift |  |
|  |  |  |  |  |
| Δ*noc*Δ*comEB* |  |  |  |  |
| #22 | 996,316 | *dnaA* | R254Q | Yes |
| #23 | 978,230 | SAOUHSC_01679: MiaB 2-methylthioadenine synthetase | Q150* | Yes |
| #24 | 1,234,711 | *trmB*: tRNA (guanine-N(7)-)-methyltransferase trmB  SAOUHSC 01866: Phosphotransferase enzyme family | deletion: 1772372- 1772564bp |  |
| #25 | 3,175,065 | SAOUHSC_02963: clfB clumping factor B | D617G |  |
| #26 | 1,658,211 | SAOUHSC_01866: Phosphotransferase enzyme family | E157G | Yes |
| #27 | 2,465,322 | SAOUHSC_01679: MiaB 2-methylthioadenine synthetase  SAOUHSC_01866: Phosphotransferase enzyme family | Q150*  L185R |  |
| #28 | 1,249,282 | SAOUHSC_00018: DnaC helicase | A280V | Yes |
| #29 | 2,025,633 | SAOUHSC_01866: Phosphotransferase enzyme family | D147G |  |
