# Supplementary material for: The nucleoid occlusion factor Noc controls DNA replication initiation in Staphylococcus aureus
Source: PLoS Genet. 2017 Jul 19;13(7):e1006908. doi: 10.1371/journal.pgen.1006908 (PMC5540599; doi:10.1371/journal.pgen.1006908)
Supplement: S4 Table — (DOCX) [file pgen.1006908.s005.docx]

**S4 Table** Strains used in this study

| **Strain** | **Relevant Genotype and Features** | **Source** |
| --- | --- | --- |
| ***S. aureus*** |  |  |
| RN4220 | *S. aureus* subsp. aureus NCTC8325 derivative; MSSA; r−m+; | Nair 2011 |
| HG003 | *S. aureus* subsp. aureus NCTC8325 with *rsbU* and *tcaR* repaired | Herbert 2010 |
| TM17 | RN4220 *geh*::pTM304; replication of pT181-ori containing plasmids in trans | Wang 2011 |
| TM18 | RN4220 ΔattBφ11::Orf5 (non-lysogenic and represses bacteriophage φ11 replication) | Wang 2011 |
| TM51 | TM18 (pTM378), transposon recipient strain | Wang 2011 |
| aTP310 | TM17 (pTP077), transposon donor strain | this work |
| aTP310 | TM17 (pTP077), transposon donor strain | this work |
| aTP315 | TM18 Δ*noc*::*spec* | this work |
| aTP317 | TM18 Δ*noc*::*spec*, (pTM378) transposon recipient strain | this work |
| aTP341 | HG003 Δ*noc*::*spec* | this work |
| aTP977 | RN4220 Δ*noc*::*spec* | this work |
| aTP411 | RN4220 Δ*rbd*::*kan* | this work |
| aTP428 | HG003 Δ*rbd*::*kan* | this work |
| aTP477 | RN4220 Δ*comEB*::*kan* | this work |
| aTP506 | HG003 Δ*comEB*::*kan* | this work |
| aTP730 | RN4220 Δ*parB*::*kan* | this work |
| aTP742 | HG003 Δ*parB*::*kan* | this work |
| aTP983 | RN4220 Δ*noc*::*spec* *geh*::pTP069 | this work |
| aTP359 | HG003 Δ*noc*::*spec* *geh*::pTP069 | this work |
| aTP431 | HG003 Δ*noc*::*spec* Δ*rbd*::*kan* *geh*::pTP069 (*^Sa^noc*) | this work |
| aTP508 | HG003 Δ*noc*::*spec* Δ*comEB*::*kan* *geh*::pTP069 (*^Sa^noc*) | this work |
| aTP510 | HG003 Δ*noc*::*spec* Δ*rbd*::*kan* | this work |
| aTP550 | HG003 Δ*noc*::*spec* Δ*comEB*::*kan* | this work |
| aTP979 | RN4220 Δ*noc*::*spec* *dnaA*^sup1^ | this work |
| aTP768 | HG003 Δ*noc*::*spec* *dnaA*^sup1^ | this work |
| aTP770 | HG003 Δ*noc*::*spec* *dnaA*^sup2^ | this work |
| aTP774/512 | HG003 Δ*noc*::*spec* Δ*rbd*::*kan* *dnaA*^sup1^ | this work |
| aTP776/522 | HG003 Δ*noc*::*spec* Δ*rbd*::*kan* *dnaA*^sup2^ | this work |
| aTP557 | HG003 Δ*noc*::*spec* Δ*comEB*::*kan* *dnaA*^sup3^ | this work |
| aTP518 | HG003 Δ*noc*::*spec* Δ*rbd*::*kan* *dnaC_A352V_* | this work |
| aTP780 | HG003 Δ*noc*::*spec* *dnaA*^sup1^ *geh*::pTP069 (*^Sa^noc*) | this work |
| aTP782 | HG003 Δ*noc*::*spec* *dnaA*^sup2^ *geh*::pTP069 (*^Sa^noc*) | this work |
| aTP811 | HG003 Δ*noc*::*spec* *geh*::pTP170 (*^Sa^noc_his_*) | this work |
| aTP813 | HG003 Δ*noc*::*spec* *geh*::pTP171 (*^Bs^noc_his_*) | this work |
| NE286 | USA300 *spa*::Tn | Fey 2013 |
| aTP394 | HG003 *spa*::Tn | this work |
| aTP403 | HG003 Δ*noc*::*spec* *spa*::Tn | this work |
| aTP851 | HG003 Δ*noc*::*spec* *dnaA*^sup1^ *spa*::Tn | this work |
| aTP853 | HG003 Δ*noc*::*spec* *dnaA*^sup2^ *spa*::Tn | this work |
| aTP959 | HG003 Δ*noc*::*spec* *geh*::pTP170 (*^Sa^noc_his_*) *spa*::Tn | this work |
| aTP961 | HG003 Δ*noc*::*spec* *geh*::pTP171 (*^Bs^noc_his_*) *spa*::Tn | this work |
| aTP821 | HG003 pLOW-FtsZ-GFP | this work |
| aTP845 | HG003 Δ*noc*::*spec*, pLOW-Zgfp | this work |
| aTP823 | HG003 Δ*noc*::*spec* *dnaA*^sup1^, pLOW-Zgfp | this work |
| aTP825 | HG003 Δ*noc*::*spec* *dnaA*^sup2^, pLOW-Zgfp | this work |
| aTP919 | HG003 pNDX2 | this work |
| aTP921 | HG003 pNDX2-*dnaA* | this work |
| aTP923 | HG003 pNDX2-*dnaA*^R318H^ | this work |
| aTP925 | HG003 *spa*::Tn, pNDX2 | this work |
| aTP927 | HG003 *spa*::Tn, pNDX2-*dnaA* | this work |
| aTP929 | HG003 *spa*::Tn, pNDX2-*dnaA*^R318H^ | this work |
| aTP949 | HG003 Δ*noc*::*spec,* pNDX2 | this work |
| aTP951 | HG003 Δ*noc*::*spec,* pNDX2-*dnaA* | this work |
| ***B. subtilis*** |  |  |
| PY79 | wild type | Youngman 1983 |
| bRB73 | PY79 Δ*noc*::*tet* | Lab stock |
| bDR3019 | PY79 Δ*soj(parA)* | Lab stock |
| bDR2292 | PY79 Δ*spo0J(parB)*::*spec* | Lab stock |
| bML712 | PY79 Δ*noc*::*tet* Δ*minD*::*kan* | Lab stock |
| bTP039 | PY79 Δ*noc*::*tet* *ycgO*::*P_spank_*- *^Bs^noc_his_* *(spec)* | this work |
| bTP041 | PY79 Δ*noc*::*tet* *ycgO*::*P_spank_*-*^Sa^noc_his_ (spec)* | this work |
| bTP043 | PY79 Δ*noc*::*tet* Δ*minD*::*kan* *ycgO*::*P_spank_*- *^Bs^noc_his_* *(spec)* | this work |
| bTP045 | PY79 Δ*noc*::*tet* Δ*minD*::*kan* *ycgO*::*P_spank_*-*^Sa^noc_his_ (spec)* | this work |
| bTP061 | PY79 Δ*noc*::*tet* *amyE*::*P_xyl_*-*^Sa^noc-yfp (spec)* | this work |
| 4702 | 168 *trpC2* Δ*noc*::*tet* *amyE*::*P_xyl_*-*^Bs^noc-yfp (spec)* | Wu 2009 |
| bRB467 | PY79 Δ*noc*::*tet* *amyE*::*P_xyl_*-*^Bs^noc-yfp (spec)* | Lab stock |
