# Supplementary material for: The nucleoid occlusion factor Noc controls DNA replication initiation in Staphylococcus aureus
Source: PLoS Genet. 2017 Jul 19;13(7):e1006908. doi: 10.1371/journal.pgen.1006908 (PMC5540599; doi:10.1371/journal.pgen.1006908)
Supplement: S5 Table — (DOCX) [file pgen.1006908.s006.docx]

**S5 Table** Plasmids used in this study

| **Plasmid** | **Relevant features** | **Source** |
| --- | --- | --- |
| pLOW-FtsZ-GFP | *S. aureus* FtsZ-GFP expression plasmid | Liew 2011 |
| pMAD | Vector for allelic replacement | Arnaud 2004 |
| pNDX2 | *S. aureus* vector containing aTc-inducible promoter | Kurokawa 2009 |
| pNDX2-dnaA | *S. aureus* aTc-inducible *dnaA* expression plasmid | Kurokawa 2009 |
| pNDX2-R318H | *S. aureus* aTc-inducible *dnaA_R318H_* expression plasmid | Kurokawa 2009 |
| pTM304 | pCL25-P_Pen_ repC11 TT | Wang 2011 |
| pTM378 | pWV01^Ts^ *ori* *aphA-3* Gram^+^ RBS HMAR1 C9 transposase | Wang 2011 |
| pTP016 | pMAD derivative | this work |
| pTP044 | L54a integrase expression plasmid | this work |
| pTP069 | *P_tet_*-*^Sa^noc S. aureus* integration plasmid | this work |
| pTP077 | Transposon containing plasmid with NotI sites | this work |
| pTP078 | Δ*noc*::*spec* insertion-deletion plasmid | this work |
| pTP083 | Δ*parB*::*kan* insertion-deletion plasmid | this work |
| pTP088 | Δ*rbd*::*kan* insertion-deletion plasmid | this work |
| pTP095 | Δ*comEB*:*kan* insertion-deletion plasmid | this work |
| pTP137 | *ycgO*::*P_spank_*- *^Bs^noc spec* | this work |
| pTP167 | *P_tet_*-(optimized RBS) *^Bs^noc S. aureus* integration plasmid | this work |
| pTP169 | *ycgO*::*P_spank_*-(optimized RBS) *^Sa^noc* *spec* | this work |
| pTP170 | *P_tet_*-*^Sa^noc_his_ S. aureus* integration plasmid | this work |
| pTP171 | *P_tet_*-(optimized RBS) *^Bs^noc_his_ S. aureus* integration plasmid | this work |
| pTP173 | *ycgO*::*P_spank_*- *^Bs^noc_his_ spec* | this work |
| pTP174 | *ycgO*::*P_spank_*-(optimized RBS) *^Sa^noc_his_ spec* | this work |
| pTP200 | *amyE*::*P_xyl_*-(optimized RBS) *^Sa^noc-yfp spec* | this work |
