# Supplementary material for: The nucleoid occlusion factor Noc controls DNA replication initiation in Staphylococcus aureus
Source: PLoS Genet. 2017 Jul 19;13(7):e1006908. doi: 10.1371/journal.pgen.1006908 (PMC5540599; doi:10.1371/journal.pgen.1006908)
Supplement: S6 Table — (DOCX) [file pgen.1006908.s007.docx]

**S6 Table** Oligonucleotides primers used in this study

| Used for plasmid construction | |
| --- | --- |
| oTP023 | GTTACGTTACACATTAACTAGACCATGGTATGGATCCATCACCGATACGCGAGCG |
| oTP024 | CGTGTGATGCGCTGCGTCCGCTACACCTCCGGATAATAAATATAT |
| oTP025 | ATATATTTATTATCCGGAGGTGTAGCGGACGCAGCGCATCACACG |
| oTP026 | CAACATAATATGCTAAAGGCCTCGGTCTTTTGCGCAGTCGGC |
| oTP027 | GCCGACTGCGCAAAAGACCGAGGCCTTTAGCATATTATGTTG |
| oTP028 | CGCTCGCGTATCGGTGATGGATCCATACCATGGTCTAGTTAATGTGTAACGTAAC |
| oTP046 | CGGCTCGAGTTCTGCTCCCTCGCTCAG |
| oTP047 | CGGGTCGACATCAGGGAGCACTGGTCAAC |
| oTP048 | CATGGTATGAATTCATAGTCGACTTACTCGAGTATGCTAGCAATCGGCCGATTG |
| oTP049 | GATCCAATCGGCCGATTGCTAGCATACTCGAGTAAGTCGACTATGAATTCATAC |
| oTP078 | CCCCGAAAAGTGCCACCTGGAATTCGACTCTCTAGCTTGAG |
| oTP083 | GTCAATTGTCTGATTCGTTACCTCCCCACACAACCAACAAAAC |
| oTP084 | GTTTTGTTGGTTGTGTGGGGAGGTAACGAATCAGACAATTGAC |
| oTP085 | CTCAAGCTAGAGAGTCGAATTCCAGGTGGCACTTTTCGGGG |
| oTP088 | GCAATTAATGTGAGTTAGGAAGCTTCATACGGCAATAGTTACCC |
| oTP089 | GCCCCGCAAAAGACATAATGGAGCTGTAATATAAAAACCTTC |
| oTP090 | GGGTAACTATTGCCGTATGAAGCTTCCTAACTCACATTAATTGC |
| oTP091 | GAAGGTTTTTATATTACAGCTCCATTATGTCTTTTGCGGGGC |
| oTP096 | TTATAAAAGCCAGTCATTAGGGCTAGCCACTCATAGTTCTAAAC |
| oTP097 | ATCATCTCAATATCCGAATAGGAAATTCAGAGAAGCCTTTGAG |
| oTP120 | CATTAACTAGACCATGGTATGCCAGAAGACTTAGATTATAGTAAG |
| oTP123 | CGTATCGGTGATGGATCCAATCGCCGGAAACGTTGCAAATATAC |
| oTP126 | ATAGTTAACAAGGAGCGAATGGATAATGAAAAAAC |
| oTP127 | AATGAATTCCTACTACTAACGTTTATATATTCGAA |
| oTP196 | ATGGCATGCCGAATTGGGCCGGCGGCCGCTCTAGTAACAGGTTGGCTG |
| oTP197 | AAAGGATCCAGACTGGACGGGCGGCCGCTCTAGTAACAGGTTGGCTG |
| oTP198 | CCGTCCAGTCTGGATCC |
| oTP199 | GGCCCAATTCGGCATGC |
| oTP200 | GAGGGAGCAGAACTCGAGCCGCTTCAATATGTCCAATGATGTCATC |
| oTP201 | GTGCTCCCTGATGTCGACCCGCGTTAGTAGTAGGATGTCGTATAC |
| oTP226 | CATTAACTAGACCATGGTATGCTGTAAAATCAACGTACTTAAATTTG |
| oTP227 | GAGGGAGCAGAACTCGAGCCGTTTTGACAATTCACTCACATCAC |
| oTP228 | GTGCTCCCTGATGTCGACCCGCGTAGGTATGGTAAATAGTTACAC |
| oTP229 | CGTATCGGTGATGGATCCAATCGGTTGAGGTGTTTTAATACCTTC |
| oTP244 | CATTAACTAGACCATGGTATGGCACCTTTGTGTTTCTCCC |
| oTP245 | GTGCTCCCTGATGTCGACCCGGGCTATTACTATTAAGTGAATCG |
| oTP246 | GAGGGAGCAGAACTCGAGCCGGTCTATGTTCATTTAGTCCTCC |
| oTP247 | CGTATCGGTGATGGATCCAATCGACGATTGTGTAGCGCATGG |
| oTP265 | CATTAACTAGACCATGGTATGGACATATTTGTCTTGGTTAGC |
| oTP266 | GAGGGAGCAGAACTCGAGCCGCATAAAATATTCTTCCCATTTG |
| oTP267 | GTGCTCCCTGATGTCGACCCGCTGACTAAAGGTTAATGTTTTGC |
| oTP268 | CGTATCGGTGATGGATCCAATCCGATAAATGAAGCTAATTGTGC |
| oTP310 | CTTGAGGGTAGCGGACAAG |
| oTP387 | ATACCCGGGAAGGAGCGAATGGATAATGAAAAAAC |
| oTP389 | ATACCCGGGAAGGTGGTGTAGGTACATG |
| oTP401 | CGAGCTAGCCTACTACTAACGTTTATATATTCGAA |
| oTP402 | CGAGCTAGCCTATTTTGGTATGCGAATCGTT |
| oTP414 | GTATGATGGTACCGTTAACAAGGTGGTGTAGGTACATG |
| oTP415 | GAAAAGTGCCACCTGGAATTCCTATTTTGGTATGCGAATCGTT |
| oTP439 | GTCCCGGGAAGGAGGAACTACTATGAAAAAACCTTTTTCAAAATTATTTGG |
| oTP440 | GAAAAAGGTTTTTTCATAGTAGTTCCTCCTTCCCGGGACAAG |
| oTP441 | GTACCGTTAACAAGGAGGAATAAAAAATGAAGCATTCATTCTCTCG |
| oTP442 | GAATGAATGCTTCATTTTTTATTCCTCCTTGTTAACGGTACC |
| oTP445 | GGTGGTGGTGGTGGTGCCCGGGACGTTTATATATTCGAATTTTTATTTCATAATAATC |
| oTP446 | GGTGGTGGTGGTGGTGCCCGGGTTTTGGTATGCGAATCGTTAATTG |
| oTP447 | GGGCACCACCACCACCACCATTAGGCTAGCTCGCATGCAAGC |
| oTP501 | AGTAGTTCCTCCTTAAGCTTGCATGCCTGCAGG |
| oTP502 | CTCGAGGGTTCCGGAGTGAG |
| oTP505 | AAGCTTAAGGAGGAACTACTATGAAAAAACC |
| oTP508 | CTCACTCCGGAACCCTCGAGACGTTTATATATTCGAATTTTTATTTCATAA |
| oTP510 | CGCCATTCGCCAGGGCTG |
| oTP511 | TGGTAGCGACCGGCGCTCAGGATCCTTATTTGTATAGTTCATCCATGCCATG |
| Used for marker frequency analysis | |
| oTP478 | CAGAAACACGCCCAGTACTAA |
| oTP479 | CTGCAACTTTCTTACAGCCAAG |
| oTP480 | GAGTATGGTGGACGTGGTATG |
| oTP481 | TTTGTACTGGCGTAGGCTTT |
| oTP482 | GGGTAGTAGGCCAGCAATTTA |
| oTP483 | CCTTGATAAGTACATGGCGATTTG |
| oTP484 | CCTCATGTACAAGACCGGTAAG |
| oTP485 | GTTCCTCCATGTTCAGCTACA |
| Confirmation of *dnaA* suppressor mutants | |
| oTP342 | CGTCCAACTCATGATTTTATAAG |
| oTP343 | GATTACATTTCCCAAAGTTTCC |
| Confirmation of insertion into *ycgO* site | |
| oKM011 | CAATTCCCGGATTCCTGG |
| oKM012 | CCACTGGCTTTTGCAGTTC |
