# Supplementary material for: The nucleoid occlusion factor Noc controls DNA replication initiation in Staphylococcus aureus
Source: PLoS Genet. 2017 Jul 19;13(7):e1006908. doi: 10.1371/journal.pgen.1006908 (PMC5540599; doi:10.1371/journal.pgen.1006908)
Supplement: S7 Table — (DOCX) [file pgen.1006908.s008.docx]

**S7 Table** Peaks enriched by ChIP-seq in *S. aureus* using anti-*^Bs^*Noc antibody

| Peak # | Peak position (bp) | FIMO identified motif position (bp) | Score^1^ | *p*-value^2^ | q-value^3^ | Matched sequence |
| --- | --- | --- | --- | --- | --- | --- |
| 1 | 67149 - 67300 | 67228 -  67241 | 15.95 | 2.03E-06 | 0.26 | ATTTCTCGGGCAAT |
| 2 | 83280 - 83445 | 83342 -  83355 | 6.058 | 8.85E-05 | 1 | ATTTCCTGGGTTAC |
| 3 | 104230 - 104382 | 104303 -  104316 | 15.95 | 2.03E-06 | 0.26 | ATTTCCCAGGCAAT |
| 4 | 128017 -  128170 | 128098 -  128111 | 11.01 | 1.53E-05 | 0.861 | ATAACCCGGAAAAT |
| 5 | 133029 -  133188 | 133107 -  133120 | 15.95 | 2.03E-06 | 0.26 | ATTGCCCAGGAAAT |
| 6 | 143823 -  143988 | 143895 -  143908 | 16.96 | 4.98E-07 | 0.128 | ATTTCCCGGGGCAT |
| 7 | 153267 -  153427 | 153339 -  153352 | 21.91 | 5.34E-08 | 0.0301 | ATGTCCCGGGAAAT |
| 8 | 169562 -  169711 | 169633 -  169646 | 21.91 | 5.34E-08 | 0.0301 | ATTTCCCGGGAATT |
| 9 | 200985 -  201140 | 201061 -  201074 | 15.95 | 2.03E-06 | 0.26 | ATATCCTGGGAAAT |
| 10 | 266582 -  266747 |  |  |  |  |  |
| 11 | 307853 -  308009 | 307912 -  307925 | 10 | 3.47E-05 | 0.981 | ATTTCCTAGGAAAA |
| 12 | 331029 -  331175 | 331107 -  331120 | 10 | 3.47E-05 | 0.981 | ATTTCCTAGGGAAT |
| 13 | 345989 -  346139 | 346071 -  346084 | 14.95 | 3.26E-06 | 0.288 | ATTTCCTCGGAAAT |
| 14 | 415983 -  416134 | 416059 -  416072 | 15.95 | 2.03E-06 | 0.26 | ATTGCCTGGGAAAT |
| 15 | 429234 -  429391 | 429295 -  429308 | 12.01 | 4.64E-06 | 0.385 | ATTTCCCGGGTGGT |
| 16 | 477582 -  477741 | 477659 -  477672 | 11.01 | 1.53E-05 | 0.861 | ATTTCCCAGGTTAT |
| 17 | 567954 -  568098 | 568017 -  568030 | 14.95 | 3.26E-06 | 0.288 | ATTTCTCAGGAAAT |
| 18 | 578431 -  578600 | 578495 -  578508 | 11.01 | 1.53E-05 | 0.861 | ATTGCCCAGGAAAA |
| 19 | 691103 -  691282 | 691185 -  691198 | 20.90 | 1.50E-07 | 0.053 | ATTTCCTGGGAAAT |
| 20 | 877608 -  877773 | 877680 -  877693 | 15.95 | 2.03E-06 | 0.26 | ATTTCCTGGGTAAT |
| 21 | 2261104 -  2261269 | 2261177 -  2261190 | 20.90 | 1.50E-07 | 0.053 | ATTTCCCCGGAAAT |
| 22 | 2272079 -  2272224 | 2272134 -  2272147 | 14.95 | 3.26E-06 | 0.288 | ATTTCCCAGTAAAT |
| 23 | 2324836 -  2324988 | 2324889 -  2324902 | 10 | 3.47E-05 | 0.981 | ATGTCCTAGGAAAT |
| 24 | 2371742 -  2371901 | 2371807 -  2371820 | 21.91 | 5.34E-08 | 0.0301 | ATTTCCCGGGAAAA |
| 25 | 2405033 -  2405205 | 2405114 -  2405127 | 16.96 | 4.98E-07 | 0.128 | ATCACCCGGGAAAT |
| 26 | 2429616 -  2429779 | 2429687 -  2429700 | 14.95 | 3.26E-06 | 0.288 | ATTTCCTAGGAAAT |
| 27 | 2436417 -  2436578 | 2436491 -  2436504 | 15.95 | 2.03E-06 | 0.26 | ATTTCCCAGGCAAT |
| 28 | 2508131 -  2508295 | 2508218 -  2508231 | 14.95 | 3.26E-06 | 0.288 | ATTTCCTGGTAAAT |
| 29 | 2511766 -  2511931 | 2511842 -  2511855 | 14.95 | 3.26E-06 | 0.288 | ATTTCCCGAAAAAT |
| 30 | 2566488 -  2566633 | 2566560 -  2566573 | 10 | 3.47E-05 | 0.981 | ATTGCTCAGGAAAT |
| 31 | 2577721 -  2577893 | 2577796 -  2577809 | 14.95 | 3.26E-06 | 0.288 | ATTTCTTGGGAAAT |
| 32 | 2587242 -  2587401 | 2587325 -  2587338 | 14.95 | 3.26E-06 | 0.288 | ATTTCCTTGGAAAT |
| 33 | 2619613 -  2619751 | 2619677 -  2619690 | 14.95 | 3.26E-06 | 0.288 | ATTTACTGGGAAAT |
| 34 | 2635932 -  2636083 | 2636010 -  2636023 | 15.95 | 2.03E-06 | 0.26 | ATTTCCCTGGAAAA |
| 35 | 2685378 -  2685536 | 2685433 -  2685446 | 20.90 | 1.50E-07 | 0.053 | ATTTACCGGGAAAT |
| 36 | 2725979 -  2726137 | 2726054 -  2726067 | 21.91 | 5.34E-08 | 0.0301 | GTTTCCCGGGAAAT |
| 37 | 2749873 -  2750038 | 2749938 -  2749951 | 21.91 | 5.34E-08 | 0.0301 | ATTTCCCGGGACAT |
| 38 | 2754594 -  2754806 | 2754679 -  2754692 | 15.95 | 2.03E-06 | 0.26 | ATTTCCTGGGACAT |
| 39 | 2771924 -  2772081 | 2771996 -  2772009 | 16.96 | 4.98E-07 | 0.128 | CTTGCCCGGGAAAT |
| 40 | 2791904 -  2792075 | 2791983 -  2791996 | 12.01 | 4.64E-06 | 0.385 | ATTTCCCGGGTGAA |
| 41 | 2808311 -  2808476 |  |  |  |  |  |

1. The score is for the strength of the match to the consensus motif.

2. The *p*-value is defined as the probability of a random sequence of the same length as the motif matching that position of the sequence with as good or better a score.

3. The q-value is defined as the false discovery rate if the occurrence is accepted as significant.

Peaks #10 and #41 did not contain a potential motif that matches the consensus motif.

Peak #9, #40, #41 were more modestly enriched and peaks #2, #4, #10, #16, #18, #23 were not enriched in the ChIP-seq experiment in which in ^Bs^Noc_his_ was expressed in *S. aureus*.
