# Supplementary figures and images for: The nucleoid occlusion factor Noc controls DNA replication initiation in Staphylococcus aureus
Source: PLoS Genet. 2017 Jul 19;13(7):e1006908. doi: 10.1371/journal.pgen.1006908 (PMC5540599; doi:10.1371/journal.pgen.1006908)

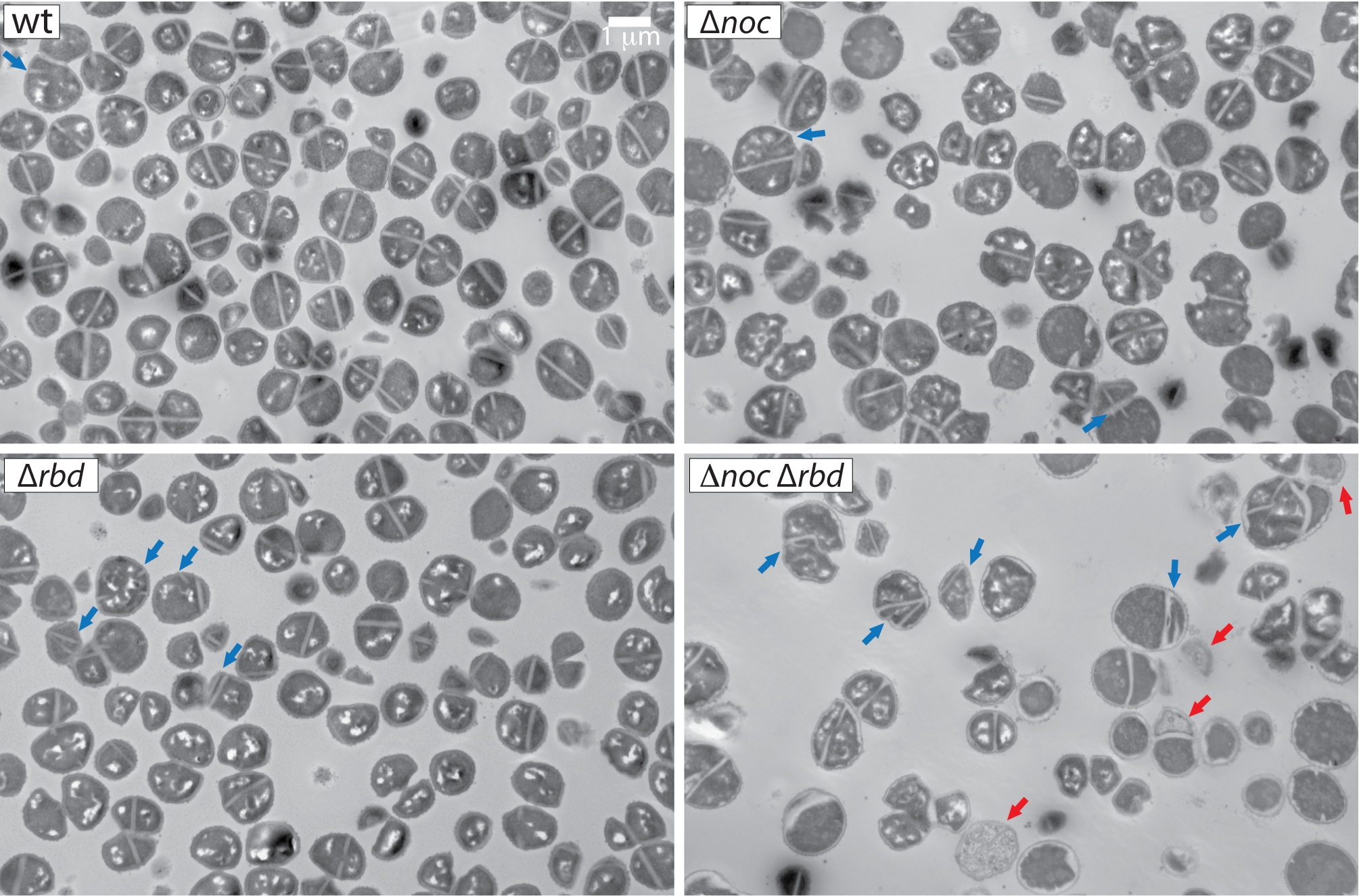

Supplement: S1 Fig — Electron micrographs of HG003 (wt) and indicated mutants. Overnight cultures were grown under permissive condition (half-strength LB without NaCl) at 30°C to an OD600 of 0.5. Cells were then washed, and diluted to an OD600 of 0.02 in LB with 0.5% NaCl, and grown at 37°C for ~4 mass doublings. Cells were then harvested, processed, and visualized by electron microscopy as described in the Methods. Examples of abnormal septa (blue arrows) and lysed cells (red arrows) are highlighted. Scale bar indicates 1 μm. (TIF) [file pgen.1006908.s010.tif]

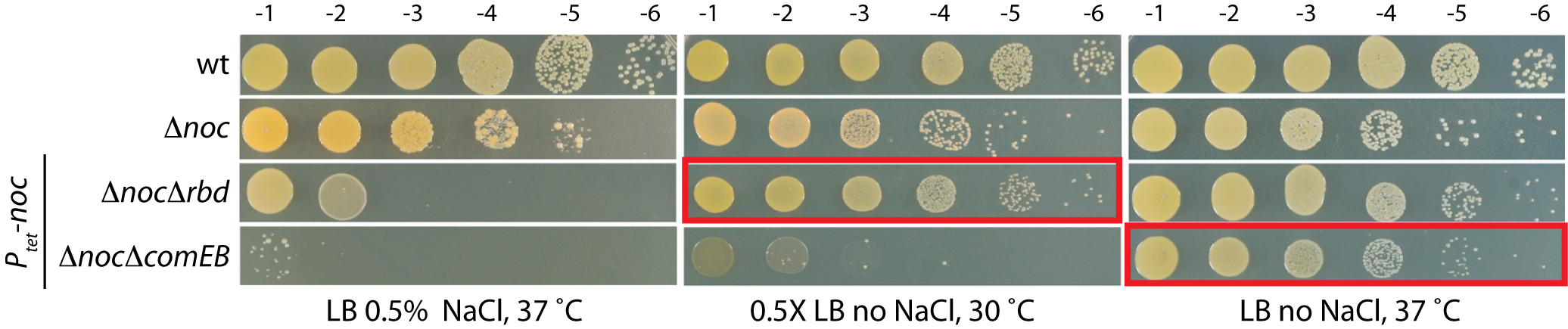

Supplement: S2 Fig — Spot dilutions of the indicated HG003 strains grown under different conditions. The Noc depletion strains (Ptet-noc) were grown overnight in liquid medium under the conditions indicated by the red boxes. Wild-type (wt) and Δnoc were grown in LB with 0.5% NaCl. Saturated cultures were washed and diluted to an OD600 of 0.2 and 10-fold serially diluted. 5 μl of each dilution were spotted onto the indicated plates, incubated overnight, and photographed. (TIF) [file pgen.1006908.s011.tif]

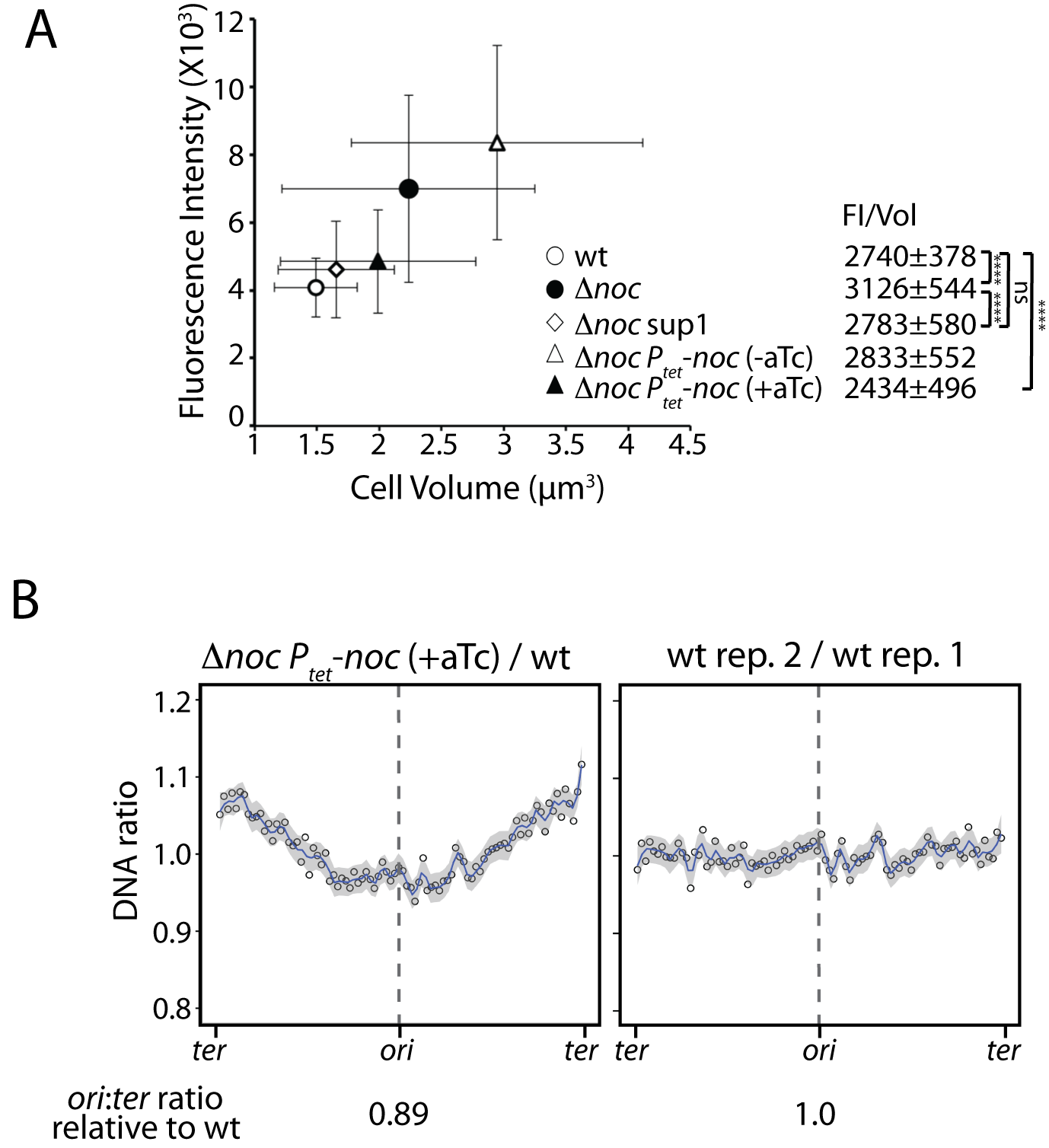

Supplement: S3 Fig — (A). Analysis of DNA content relative to cell volume in wt and derivatives. The indicated strains grown under the same conditions as described in Fig 4B were fixed with ethanol and later stained with the fluorescent DNA dye propidium iodide (PI) and examined by phase contrast and fluorescence microscopy. The Fluorescence intensity (mean ± standard deviation) and cell volume (mean ± standard deviation) were quantified from fluorescence and phase contrast images (n>100), and plotted on the graph. The ratio of fluorescence intensity to cell volume (FI/Vol) of each strain is shown. p ≤ 0.0001 (****) p > 0.05 (ns). (B). The plots show the ratios of the indicated genomic profiles. Overnight cultures of S. aureus strain RN4220 (wt) and Δnoc cells expressing noc under the control of the Ptet promoter were diluted to OD600 = 0.01 and grown in TSB medium without or with inducer (aTc) at 37°C. Genomic DNA was isolated after 5 mass doublings and analyzed by whole-genome sequencing. The total sequencing reads from each strain were normalized to 51 million and the data were plotted relative to the sequencing reads of the first biological replicate of wild-type. Circles show 30 kb bins. Blue lines and grey area represent the smoothed conditional mean and 95% confidence band for the regression curve, respectively (spanS = 0.08). The data from one of two biological replicates are shown. The left plot is identical to the one presented in Fig 4C and is included to facilitate a direct comparison. (TIF) [file pgen.1006908.s012.tif]

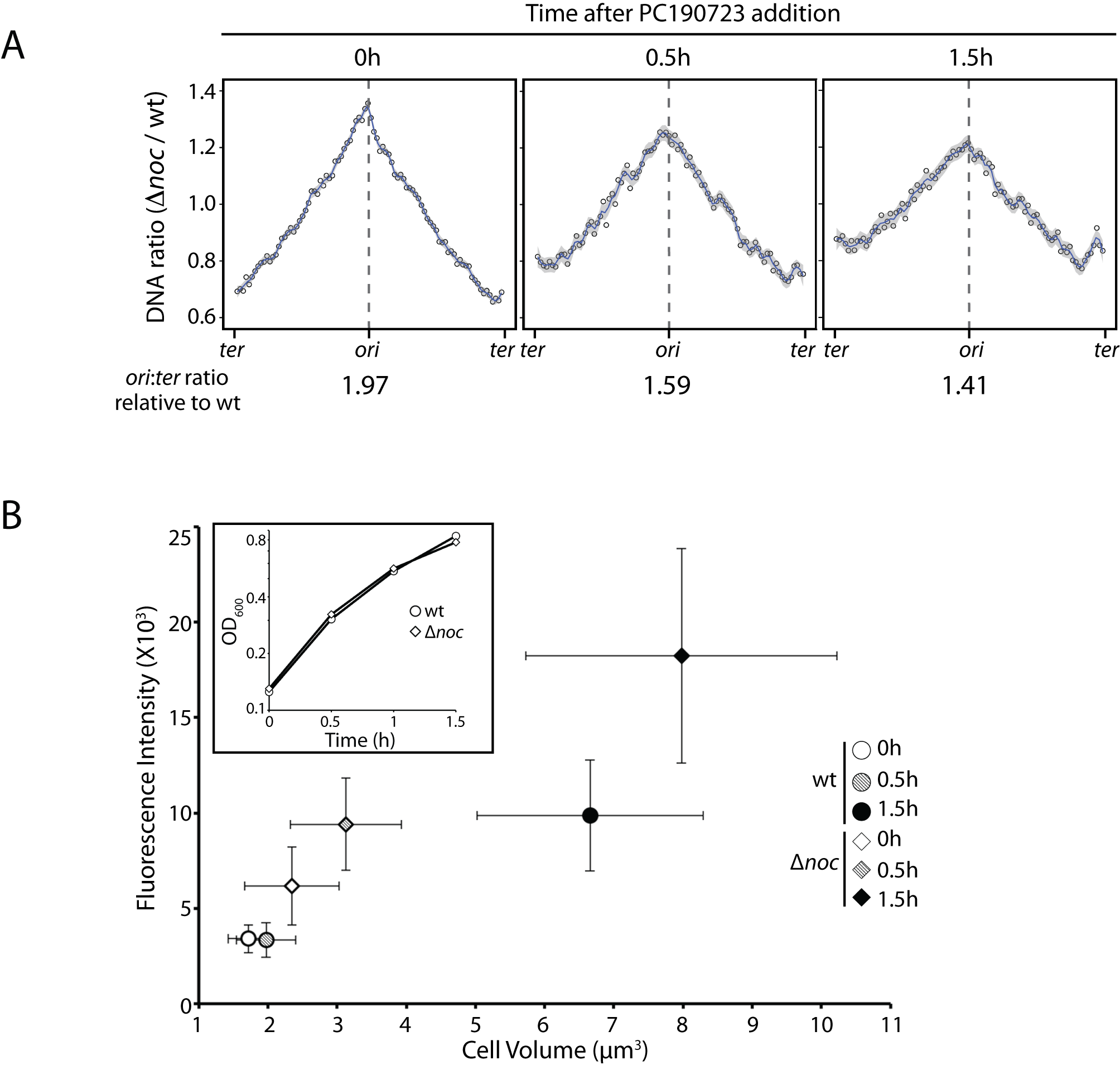

Supplement: S4 Fig — (A). The plots show the ratios of genomic profiles from Δnoc and wild-type (wt) before and after treatment with the FtsZ inhibitor PC190723. Overnight cultures of S. aureus strain RN4220 (wt) and Δnoc were diluted to OD600 ~ 0.01 and grown in TSB medium at 37°C. The inhibitor PC190723 (2μg/ml) was added to each culture at OD600 = 0.13. Cultures were harvested before (0h) and 0.5h or 1.5h following the addition of the drug. The total sequencing reads from each strain were normalized to 51 million and the data were plotted as a ratio of Δnoc to wild-type at each time point. Circles show 30 kb bins. Blue lines and grey area represent the smoothed conditional mean and 95% confidence band for the regression curve, respectively (spanS = 0.08). The data from one of two biological replicates are shown. (B). Increase in DNA content and volume after treatment with PC190723. Cells treated the same way as in (A) were fixed with ethanol and later stained with fluorescent DNA dye propidium iodide (PI) and examined by phase contrast and fluorescence microscopy. The Fluorescence intensity (mean ± standard deviation) and cell volume (mean ± standard deviation) were quantified from fluorescent and phase contrast images (n>100), and plotted on the graph. Inset shows the growth curves for the cells used for the cytological analysis. PC190723 was added at time 0h. (TIF) [file pgen.1006908.s013.tif]

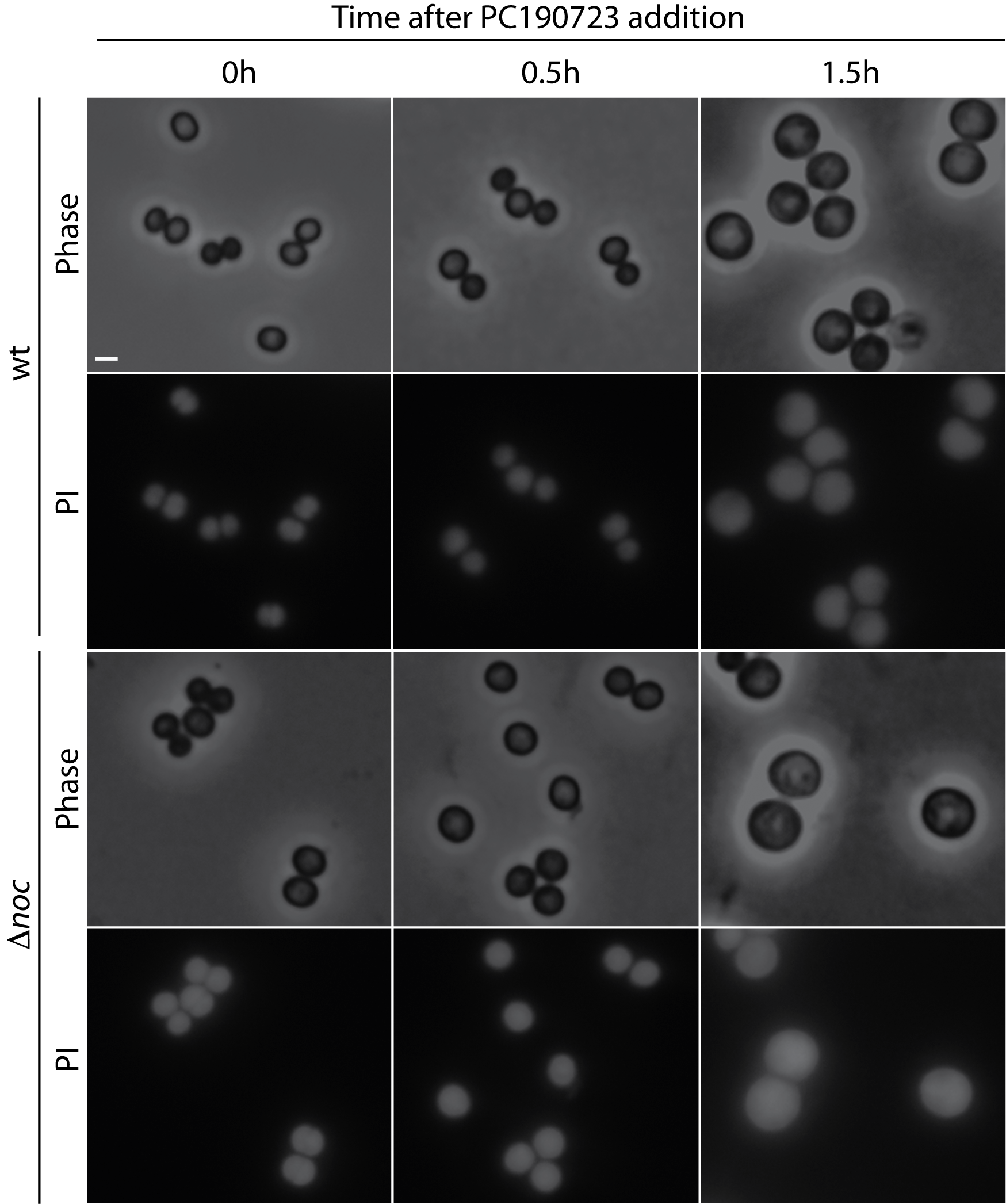

Supplement: S5 Fig — Cultures were harvested before (0h) and at 0.5h or 1.5h following the addition of PC190723 (2μg/ml), fixed with ethanol and later stained with fluorescent DNA dye propidium iodide (PI). Cells were then visualized by phase contrast (Phase) and fluorescence (PI) microscopy. Scale bar indicates 1 μm. (TIF) [file pgen.1006908.s014.tif]

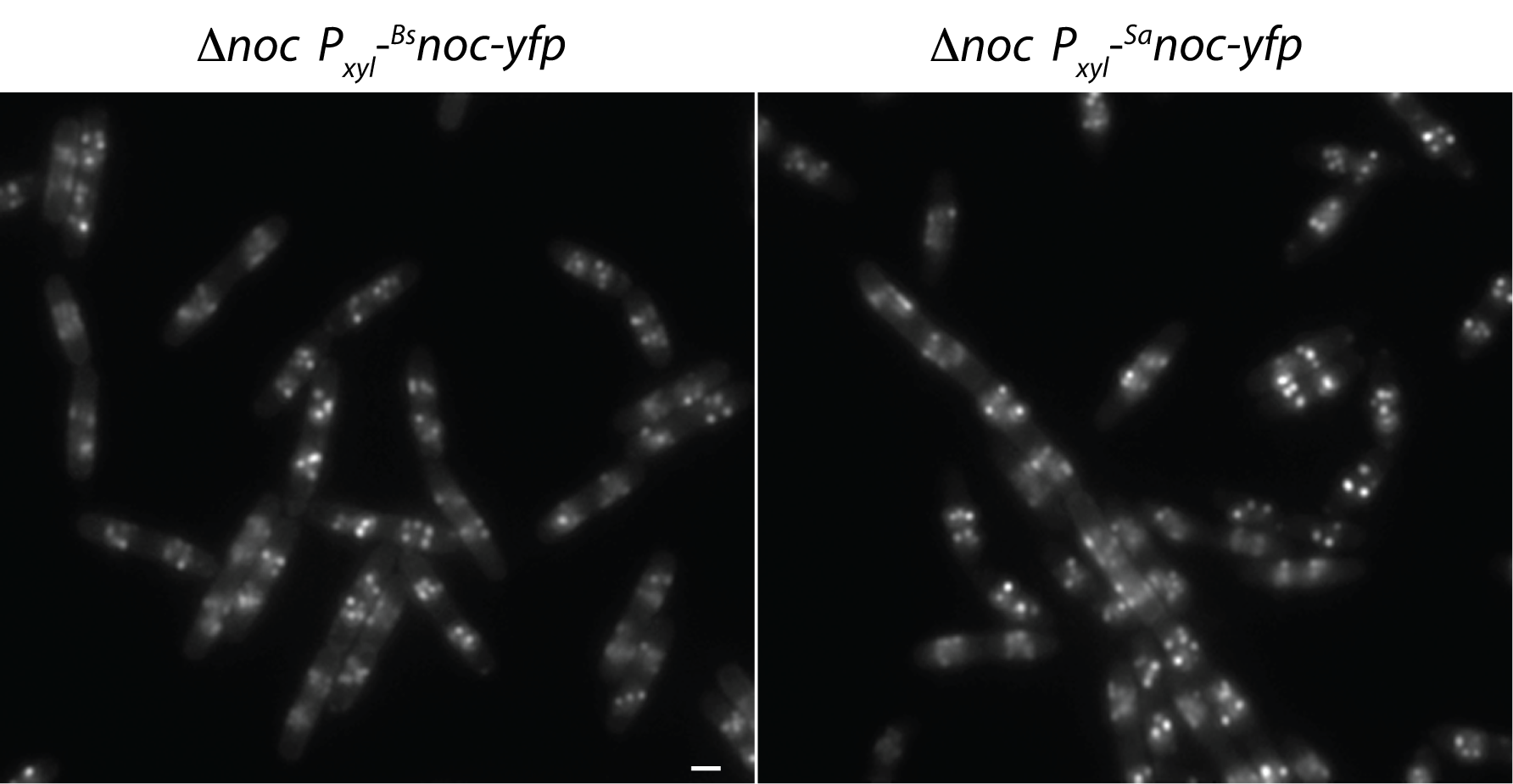

Supplement: S6 Fig — Larger fields of B. subtilis Δnoc mutants with Bsnoc-yfp or Sanoc-yfp expressed under the control of a xylose-inducible promoter. Cells were induced at OD600 = 0.01 with 0.5% xylose and analyzed by fluorescence microscopy at OD600 = 0.25. Scale bar indicates 1 μm. (TIF) [file pgen.1006908.s015.tif]

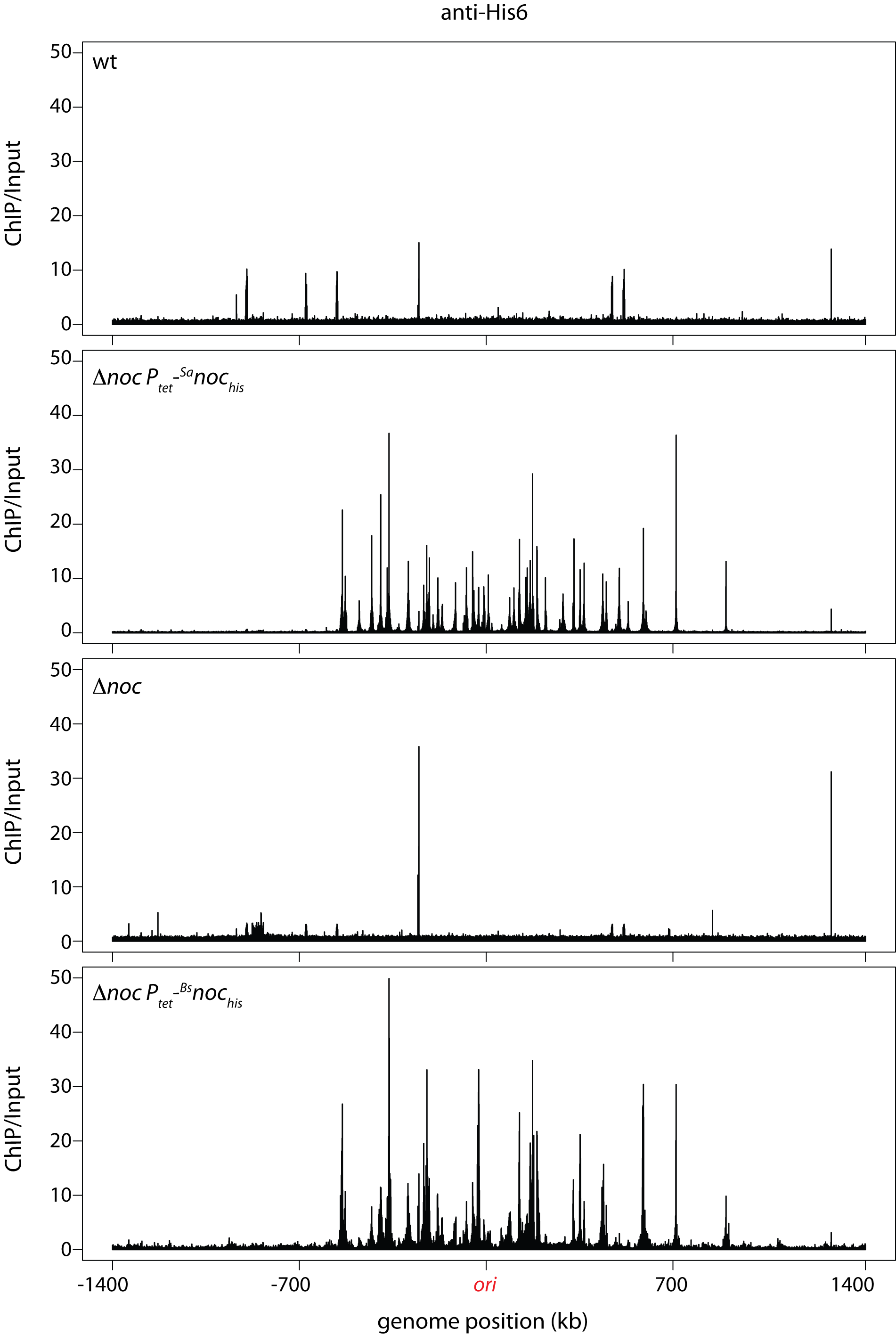

Supplement: S7 Fig — The indicated S. aureus strains were grown to OD600 = 0.4, and processed as described in the Methods. Δnoc (HG003) strains harboring Ptet fusions to his-tagged B. subtilis noc (Bsnochis) or S. aureus noc (Sanochis) were induced at OD600 = 0.05 and treated with formaldehyde at an OD600 = 0.4. Wild-type (wt) (HG003) and Δnoc were grown without inducer but were cross-linked and processed identically to the his-tagged strains. ChIP-seq was performed with anti-6XHis antibodies. The profiles were generated by normalizing the reads from the ChIP-Seq samples to those from input genomic DNA. The data are plotted in 1 kb bins. The higher background and higher peaks in the BsNochis ChIP-seq profile are due to fewer and sparser sequencing reads in this sample and the normalization process used. The origin is at position 1 with positions on the left and right chromosome arms indicated as negative or positive numbers, respectively. (TIF) [file pgen.1006908.s016.tif]

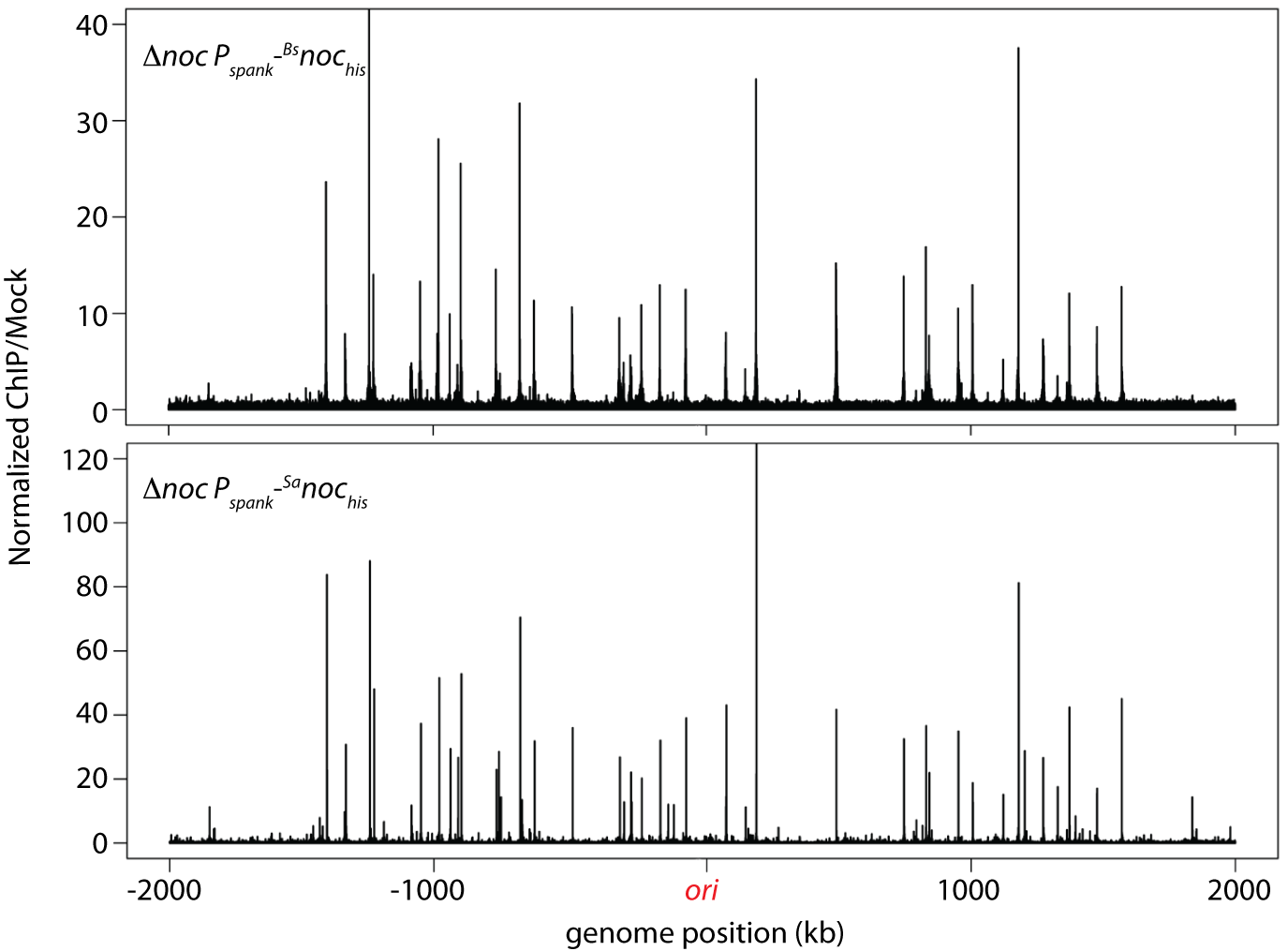

Supplement: S8 Fig — ChIP-seq using the anti-6xHis antibodies was performed on B. subtilis Δnoc cells expressing Bsnochis or Sanochis under the control of the Pspank promoter. Wild-type and Δnoc cells lacking a his-tagged noc fusion were used as negative controls. Data presented were first normalized to the total number of reads then relative to the input for each strain and finally relative to that of the relevant control sample (wild-type cells for Bsnochis and Δnoc cells for Sanochis). The data are plotted in 1 kb bins. Genome positions are labeled as in S7 Fig. (TIF) [file pgen.1006908.s017.tif]

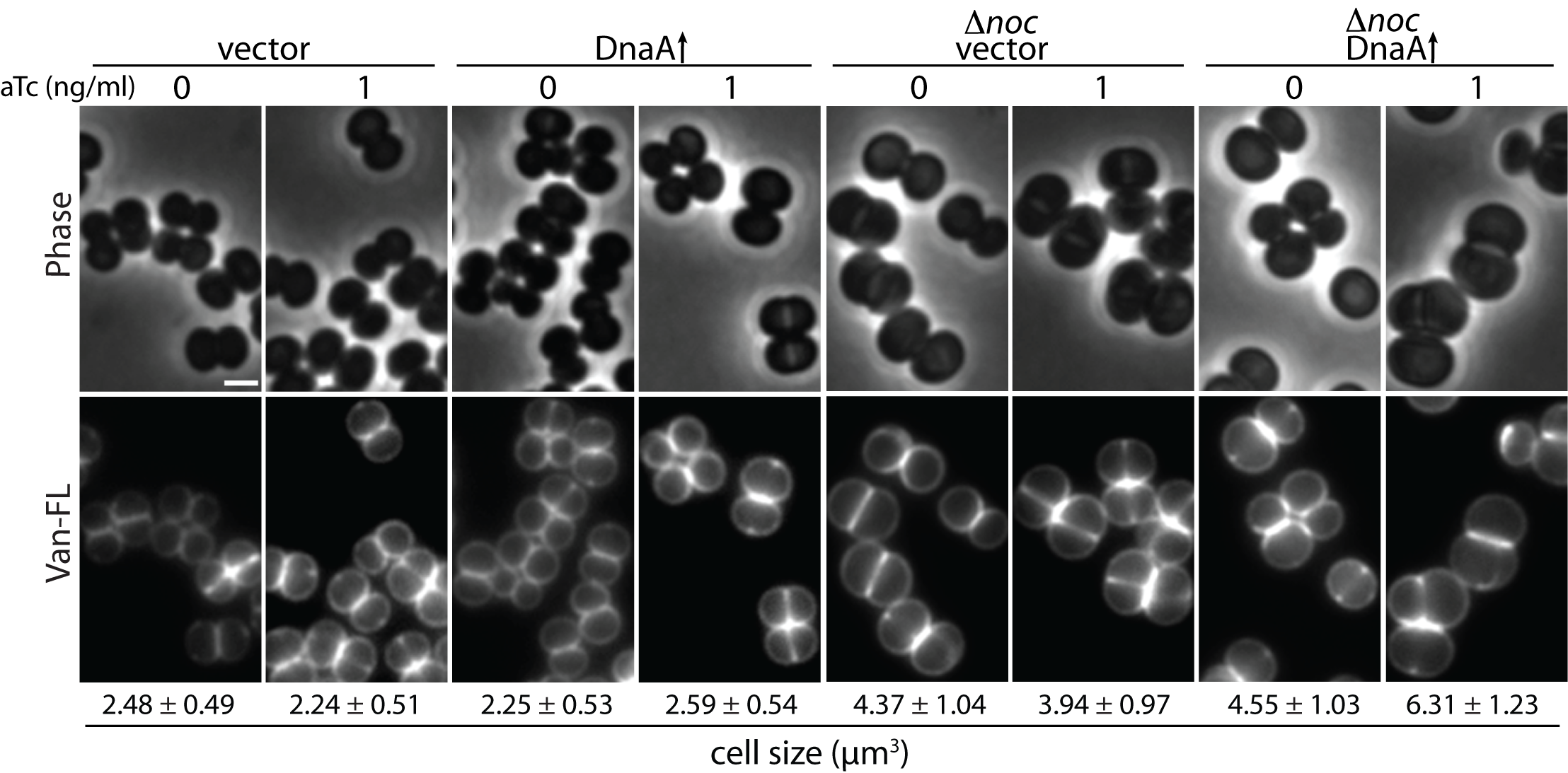

Supplement: S9 Fig — Representative fields of wild-type (HG003) or Δnoc strains harboring an empty vector or the same plasmid with dnaA fused to a Ptet promoter. Overnight cultures were diluted to an OD600 = 0.01 then grown to an OD600 of 0.05 at which time the indicated amount of inducer was added. Cells were harvested at OD600 = 0.4, washed and resuspended in phosphate buffered saline (1XPBS) with BODIPY FL-Vancomycin (Van-FL), and examined by phase contrast (Phase) and fluorescence (Van-FL) microscopy. Scale bar indicates 1 μm. Average cell volumes (μm3) ± standard deviations (n>200) are indicated below the micrographs. (TIF) [file pgen.1006908.s018.tif]

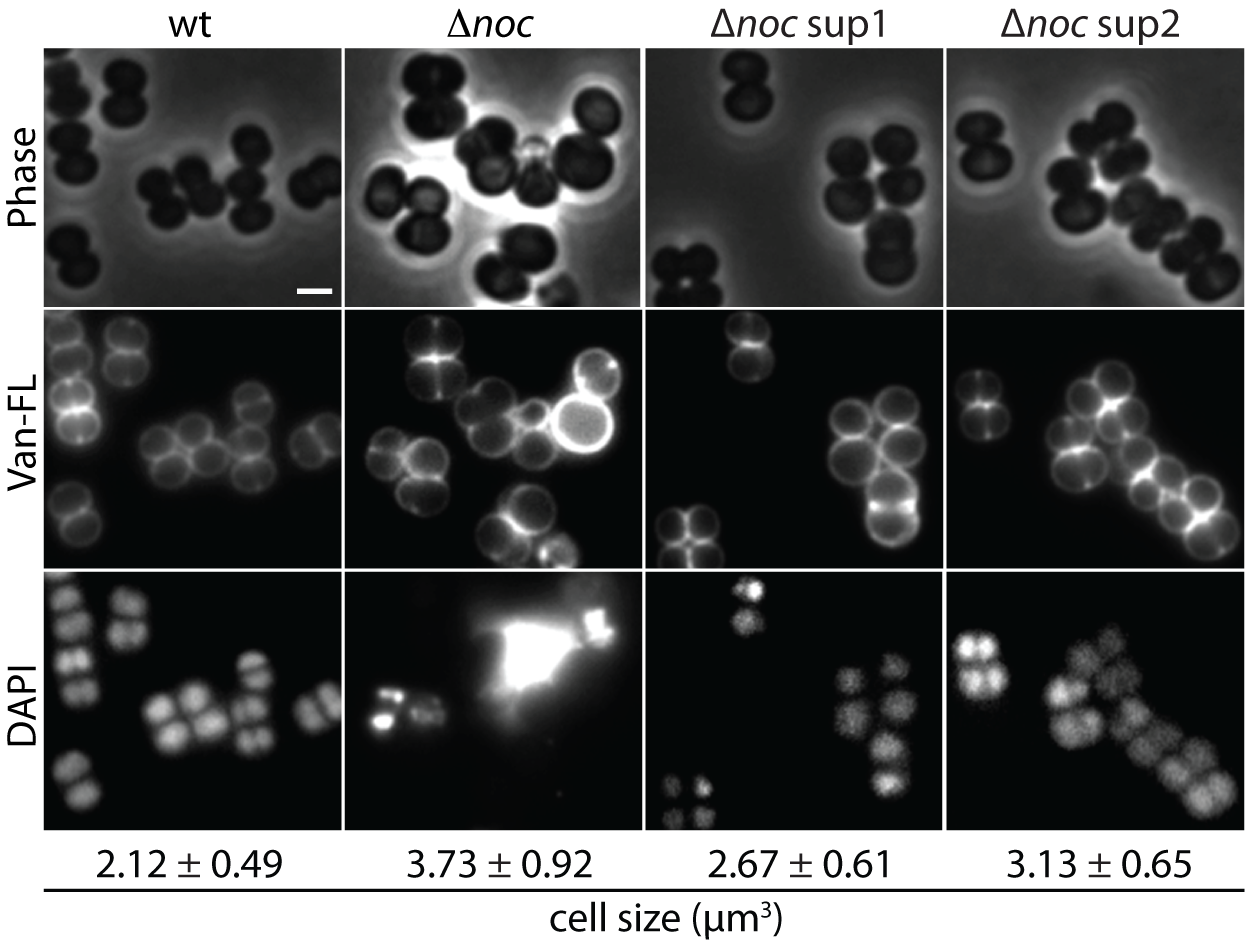

Supplement: S10 Fig — Representative fluorescent images of HG003 (wt) and indicated derivatives. Overnight cultures were diluted to OD600 = 0.01 in LB 0.5% NaCl at 37°C. When cultures reached OD600 = 0.4, cells were washed and resuspended in Phosphate buffered saline (1XPBS) with the BODIPY FL-Vancomycin (Van-FL) and DAPI and examined by phase contrast (Phase) and fluorescence microscopy. Scale bar indicates 1 μm. Average cell volumes (μm3) ± standard deviations (n>120) are shown below the micrographs. DAPI-stained cells contain a representative example of a strong-staining Δnoc cell that suggested increased cell envelope permeability in the mutant. (TIF) [file pgen.1006908.s019.tif]

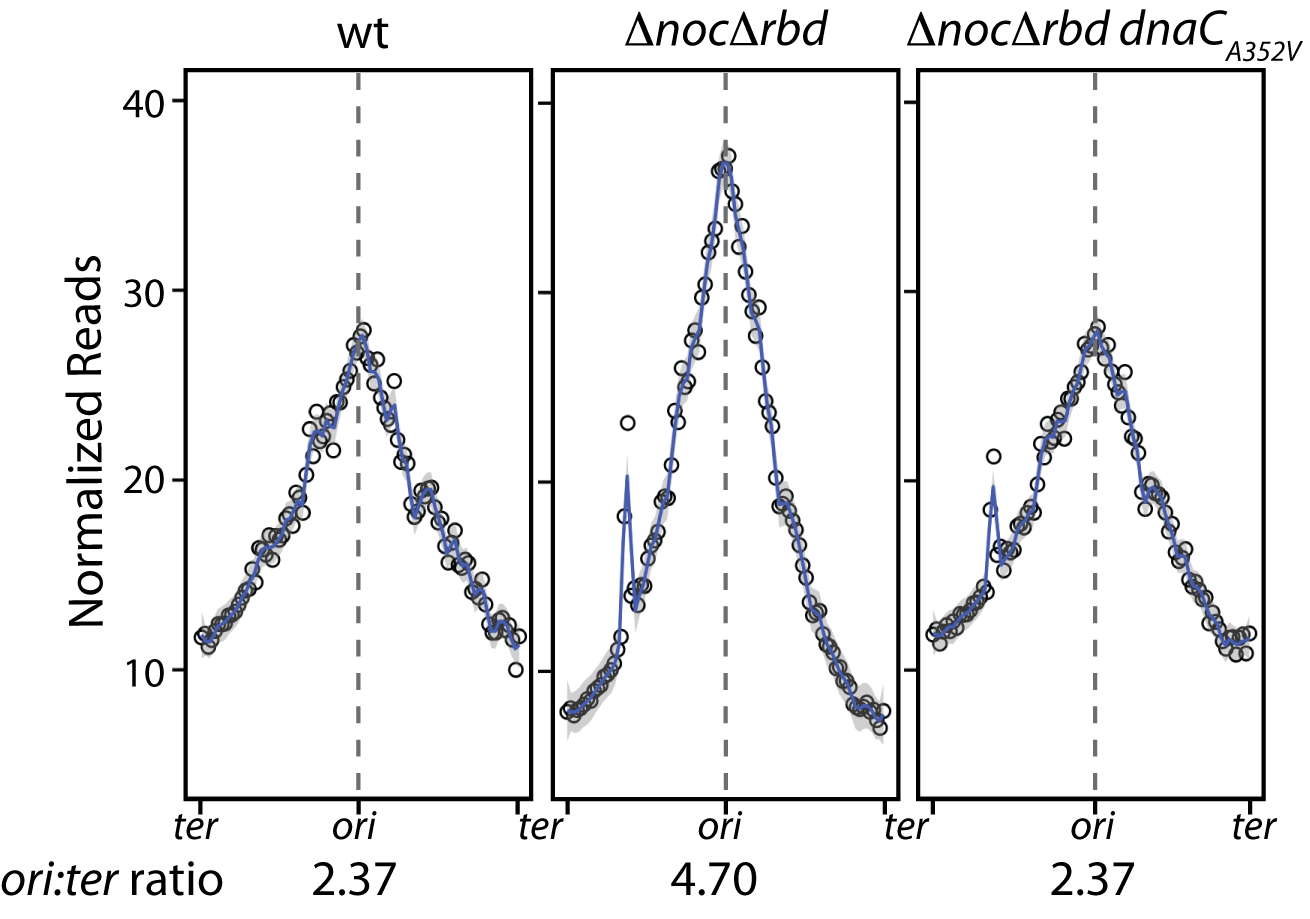

Supplement: S11 Fig — Genome-wide DNA content of the indicated strains. Overnight cultures of S. aureus strains HG003 (wt) and ΔnocΔrbd dnaCA352V were diluted to OD600 = 0.01 and grown in LB 0.5% NaCl medium at 37°C. The ΔnocΔrbd strain was first grown under permissive conditions (0.5X LB no NaCl, 30°C) to OD600 ~0.4, then back diluted into nonpermissive conditions (LB 0.5% NaCl medium, 37°C) at an OD600 ~0.04. All cultures were harvested at OD600 ~0.4. Genomic DNA was isolated and analyzed by whole-genome sequencing as described in Fig 4B. Data were normalized to 51 million reads and plotted in 30 kb bins (circles). Blue lines and grey area represent the smoothed conditional mean and 95% confidence band for the regression curve, respectively (spanS = 0.08). The data from one of two biological replicates are shown. ori:ter ratios indicated below the plots were determined using the 30kb bins spanning the origin and terminus. (TIF) [file pgen.1006908.s020.tif]
